# Mutually Exclusive Interactions of Rifabutin with Spatially Distinct Mycobacterial Cell Envelope Membrane Layers Offer Insights into Membrane-Centric Therapy of Infectious Diseases

**DOI:** 10.1021/acsbiomedchemau.2c00010

**Published:** 2022-03-24

**Authors:** Anjana P. Menon, Wanqian Dong, Tzong-Hsien Lee, Marie-Isabel Aguilar, Mojie Duan, Shobhna Kapoor

**Affiliations:** † Department of Chemistry, Indian Institute of Technology Bombay, Mumbai 400076, India; ‡ IITB-Monash Academy, Indian Institute of Technology Bombay, Mumbai 400076, India; § Innovation Academy for Precision Measurement Science and Technology, Chinese Academy of Sciences, Wuhan 430071, China; ∥ Department of Biochemistry & Molecular Biology, Monash University, Clayton, VIC 3800, Australia; ⊥ Graduate School of Integrated Sciences for Life, Hiroshima University, Hiroshima 739-8528, Japan

**Keywords:** infectious diseases, bacterial lipids, membranes, membrane−drug interactions, membrane-disruptive agents, drug discovery, biophysics, molecular modeling

## Abstract

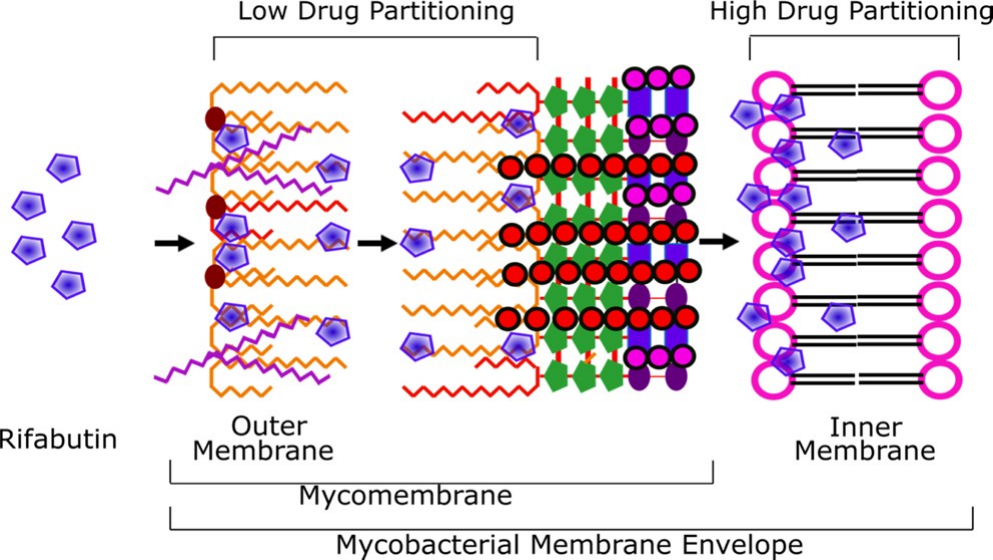

The mycobacterial cell envelope has spatially resolved inner and outer membrane layers with distinct compositions and membrane properties. However, the functional implication and relevance of this organization remain unknown. Using membrane biophysics and molecular simulations, we reveal a varied interaction profile of these layers with antibiotic Rifabutin, underlined by the structural and chemical makeup of the constituent lipids. The mycobacterial inner membrane displayed the highest partitioning of Rifabutin, which was located exclusively in the lipid head group/interfacial region. In contrast, the drug exhibited specific interaction sites in the head group/interfacial and hydrophobic acyl regions within the outer membrane. Altogether, we show that the design of membrane-active agents that selectively disrupt the mycobacterial outer membrane structure can increase drug uptake and enhance intracellular drug concentrations. Exploiting the mycobacterium-specific membrane–drug interaction profiles, chemotypes consisting of outer membrane-disruptive agents and antitubercular drugs can offer new opportunities for combinational tuberculosis (TB) therapy.

## Introduction

Tuberculosis (TB), caused by bacterium *Mycobacterium tuberculosis* (*Mtb*), represents the third most common infectious disease in the world.^1^ Several drug regimens are used to contain TB, but the emergence of multidrug resistance warrants the design and development of alternative therapeutic approaches.^2^ While lipid biosynthesis inhibitors have been explored, the biomembranes also represent potential therapeutic targets, but their role in drug interaction and resistance remains understudied.

**Figure 1:**
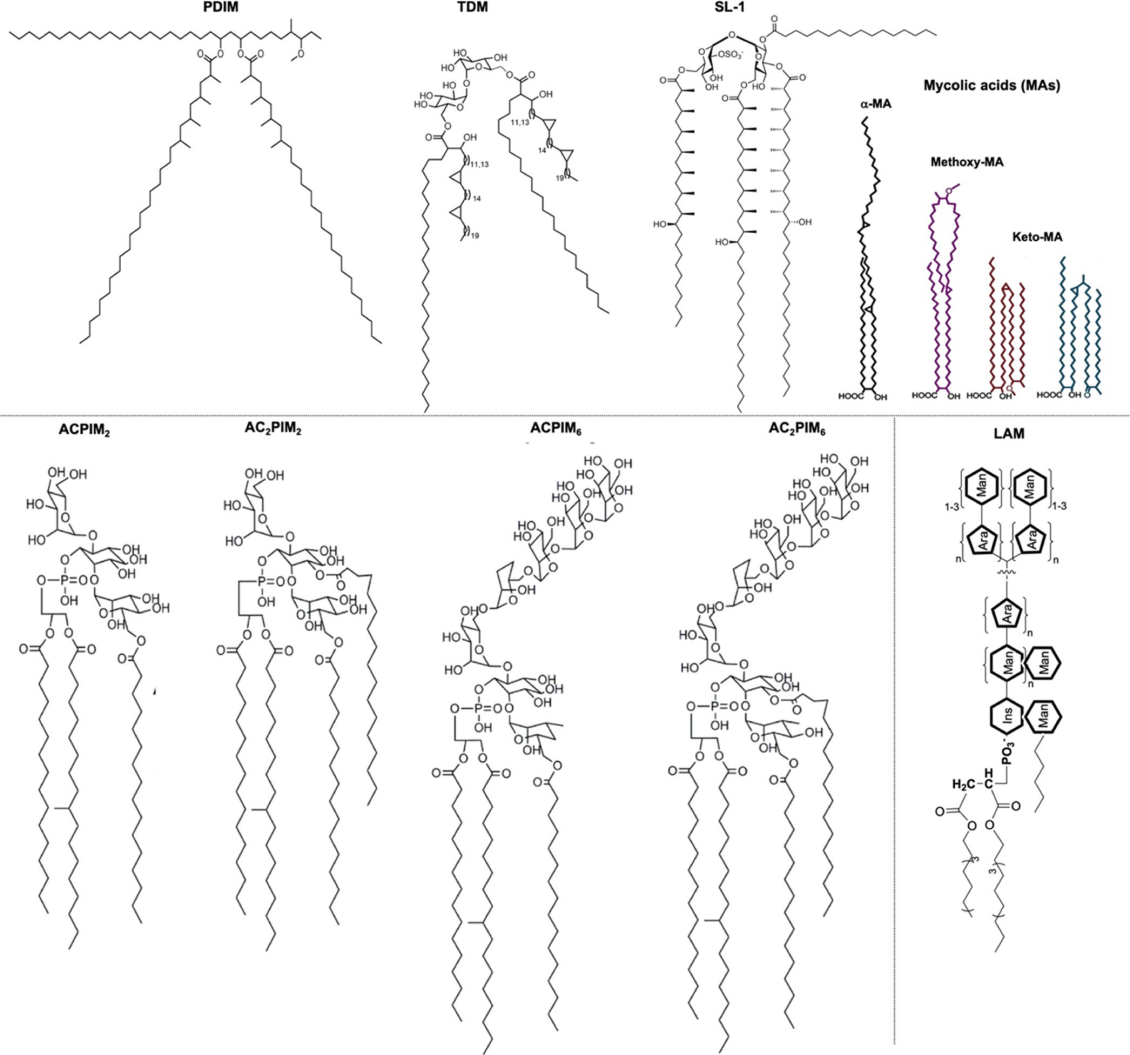
Schematic representation of the structure of the key lipids present in the mycobacterial cell envelope layers. SL-1, sulfolipids; TDM, trehalose dimycolate; PDIM and MA, noncovalent lipids in the outer membrane. The inner membrane consists of tetra- and monoacylated phospho-*myo*-inositol dimannosides (ACPIM_2_, AC_2_PIM_2_) and higher-order phosphomannosides such as ACPIM_6_ along with standard phospholipids including phosphatidylinositol (PI) and phosphatidylethanol (PE) amine. Lipoarabinomannans (LAMs) with the outer membrane-free lipids constitute the mycomembrane.

The *Mtb* cell envelope is spatially organized into an inner and outer membrane with distinct compositions.^3,4^ The main lipid compositions of the outer membrane consist of long-chain mycolic acids (MAs, C60–C90), phthiocerol dimycocerosate (PDIM), trehalose dimycolate (TDM), sulfoglycolipids (SL), phosphatidylinositol mannosides (PIMs), lipoarabinomannan (man-LAM), phenolic glycolipid (PGL), and diacylglycerol (DAG). In contrast, the lipids in the inner membrane are composed of tetra-acylated phospho-*myo*-inositol dimannosides (AC_2_PIM_2_), phosphatidylinositol mannosides (PIM6), and other ACPIMs along with phospholipids. Outer membrane lipids together with LAM and peptidoglycan-associated lipids (PALs), mainly mycolic acids (MAs) covalently bound to peptidoglycan, constitute the asymmetric mycomembranes (Figure 1).

To systematically develop strategies to target lipid membranes for enhanced uptake or disruption, a detailed understanding of drug–membrane interactions, which are lipid composition specific, is required. Various membrane properties, e.g., head group and acyl chain structure conformation, hydrophobicity, and stereochemistry, influence drug passage through the membrane,^5−8^ in addition to membrane proteins. The contribution of proteins pores and channels unique to inner and outer membranes in drug uptake remains less explored. This is partially due to both poorly defined mycobacterial membrane proteomes and the absence of proteins in model membranes. However, the absence of resistance in membrane transport proteins in *Mtb* indicates a substantial involvement of membranes in regulating passive drug movement across.^9^ In this context, the highly complex lipid repertoire and the fact that many drugs target the *Mtb* cell envelope^9,10^ make the understanding of drug–membrane interaction landscape in *Mtb* imperative. The structural differences between mycobacterial and mammalian lipids present an opportunity to develop agents that specifically target mycobacterial membranes and minimize nonspecific membrane toxicity.^7^

Although several studies have determined how drugs interact with membranes,^11−15^ the role of the complexity of the lipid composition and distribution within mycobacterial cell envelope layers in drug interaction is still missing. Using *Mycobacterium smegmatis* (*Msm*) as a model of *Mtb* and the derived protein-free model membranes, we reveal how the distinct cell membrane layers of *Msm* regulate their interaction with the antimicrobial drug, Rifabutin. We reveal the existence of a Rifabutin transbilayer gradient with a distinct degree of drug partitioning within the inner and outer layers that depend on the structural and chemical nature of the interacting lipids in these layers. The results suggest that bacteria containing multiple envelope layers may regulate their interaction with drugs and host effectors by modulating their lipidome, thereby dictating the pharmacological activity. The absence of membrane proteins and lipid asymmetry (found in native mycobacterial cell envelope) and their plausible contributions to the drug interaction landscape remains unknown at present and is attributed to the use of simplified protein-free model membranes in this work. Nonetheless, our findings will impact future studies targeted toward development of membrane lipid-centric therapeutic approaches to tackle TB by exploiting the membrane–drug interaction landscape unique to mycobacteria.

## Results and Discussion

### Design and Modeling of Model Membrane Mimetics of Mycobacterial Lipid Membranes

Protein-free lipid fractions from the *Msm* cell envelope layers (i.e., inner, outer, and peptidoglycan-associated layers) were extracted using a modified version of previous protocols.^3,4^ The modification included an additional column chromatography step for the removal of sulfosuccinic acid 1,4-bis(2-ethylhexyl) ester (AOT), otherwise used for reverse micellar solution-based selective extraction of outer membrane lipids. Thin-layer chromatography (TLC) and mass spectrometric lipidomic characterization revealed selective extraction and identification of characteristic inner, outer, and peptidoglycan-associated lipids and LAM (Figure S1 and Table S1). All of the extracts were reconstituted into model membranes in vitro, reflective of the inner, outer, and mycomembrane layers. Mycomembrane constitutes the mixed fraction of outer and peptidoglycan-associated lipids along with LAM. Based on the identity profile and rough estimate of their abundance within cell envelope layers,^3,16,17^ we developed all-atom molecular models for *Msm* inner and mycomembrane layers. The composition of the modeled membrane was inspired by the previous reports^3,4,10,16,17^ and hence should be cautiously considered as only a first approximation of the mycobacterium membranes, needing improvisions driven by improved quantitative lipidomic characterization. Nonetheless, these models recapitulated our current and previous experimental findings on their membrane properties. For instance, *Msm* inner and mycomembrane layers exhibited lipid phase segregation with variable membrane heights (total bilayer height (*h*) of 7–8 nm and Δ*h* of 1–3 nm) in excellent agreement with this study and previous reports.^4,18,19^ Second, the average order parameter (⟨*S*_CD_⟩) in the hydrophobic acyl tail regions of lipids was higher in the mycomembrane model (Table S2), as seen for MA (≈C25–37), SL-2 (≈C16–32), and TDM (≈C25–32). These agree with and provide a molecular understanding of our previous 1,6-diphenyl-1,3,5-hexatriene (DPH) anisotropy results,^4^ wherein a higher anisotropy was observed in mycomembranes compared to the inner membrane.

Though these mycobacterial mimetic model membranes recapitulate the lipid diversity inherent within different cell envelope layers and probably constitute improved model systems to investigate various aspects of mycobacterial membrane layers, there are certain limitations, which need attention. First, though, *Msm* has served as an invaluable surrogate for *Mtb* biology over many decades, much of the results need cautious interpretation. However, to our advantage, similarity within the lipidome of both the species has been demonstrated (Table S3^10^); also, recent work showed only ∼21–26% altered abundance of specific lipid molecules using comparative lipidomics.^10,20,21^ These probably correspond to lipids associated strongly with virulence. Thus, *Msm* at the moment serves as an appropriate model to investigate lipids and associated membrane properties of *Mtb*. Second, these systems lack lipid asymmetry, particularly specific to the mycomembrane, and in the future, development of protocols for generation of asymmetric lipid bilayers composed of *Msm* lipids should be attempted. Further, current knowledge about the native lipid organization in mycobacterial cell envelope is not fully known, but an agreement between the bilayer height and the existence of domains between our work and previous reports implies a near-native lipid organization. However, lipid identity within domains would need experimental verification. Finally, these systems are protein-free and hence the contribution of the same on interactions with host and drugs cannot be ascertained as of now.

Nonetheless, the models described above represent realistic models capturing lipid complexity and selectively reflective in *Msm*/*Mtb* membrane layers and knowledge of the limitations provides opportunities to develop better mycobacterial membrane models to fully capture the native membrane arrangement.

### Partitioning of Rifabutin into Mycobacterial Cell Envelope Membrane Layers

**Figure 2:**
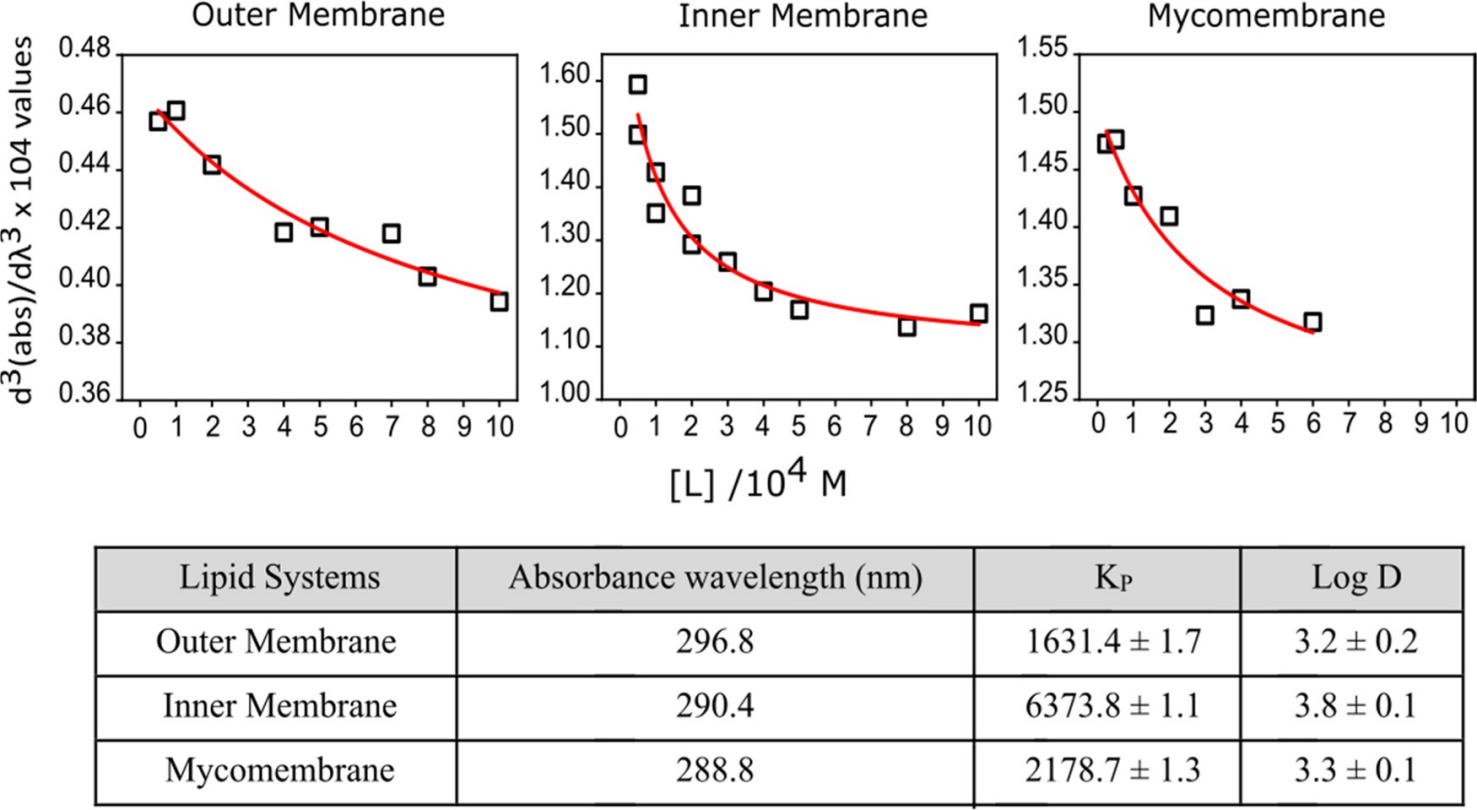
Nonlinear least-squares regression curves of the partitioning of 25 μM Rifabutin into different *Msm* membranes obtained from the third derivative UV absorption intensities ranging from 284 to 297 nm measured using UV–visible spectroscopy. Below: partition coefficient (*K*_p_) and distribution coefficient (log *D*) of Rifabutin within different *Msm* membrane systems calculated from the fitted curves.

The lipophilicity of Rifabutin governs its high propensity to interact with and distribute within the lipid membrane layers of the mycobacterial cell envelope.^22^ Given the lipidomic differences in the mycobacterial inner and outer membranes,^3,4,17^ we determined the partition coefficient (*K*_p_/log *D*) of Rifabutin in model membranes reconstituted with lipids extracted from *Msm* outer and inner layers, which have been previously characterized.^4^*K*_p_/log* D* values were calculated using derivative UV–vis spectroscopy, wherein the spectral characteristics (λ_max_) of Rifabutin changes when it transfers from the aqueous to the lipid medium, enabling quantification of its distribution between each phase. Furthermore, the use of the derivative method results in an improved resolution of the overlapped bands by the elimination of the lipid light scattering interference.^12^ Rifabutin exhibited the highest partitioning into the inner membrane (Figures 2 and S2 and Table S4). Addition of LAM and PAL to the outer membrane extract, referred to as mycomembrane (i.e., PAL and LAM associated with the outer membrane), had no major effect on drug partitioning.

In this work, all-atom molecular simulations on the *Msm* inner and outer/mycomembrane revealed a high localization of Rifabutin within the inner membrane in the interfacial lipid head group region (Movies S1 and S2), as compared to a lower but rather uniform distribution within the interfacial and hydrophobic acyl chain regions of the mycomembrane systems (Movies S3 and S4).

Rifabutin has also previously been shown to exhibit a similar partitioning into the *Msm* outer membrane and eukaryotic membranes,^7^ and overall, these nonspecific membrane interactions may underpin the toxicity observed with Rifabutin.

### Depth-Dependent Quenching of Rifabutin Reveals Specific Interaction Sites within the Mycobacterial Cell Envelope Layer

Rifabutin’s preferential interaction sites within *Msm* membranes were identified by quenching of lipid probes known to be located at defined depths within the lipid bilayer. 1,6-Diphenyl-1,3,5-hexatriene (DPH) and its trimethylammonium substitute (TMA–DPH) are located in the deep hydrophobic and interfacial regions, respectively; TMA–DPH resides at the relatively polar lipid/water interface due to its charge.^7,23^ The proximity of Rifabutin to the fluorescent probes determines the extent of their quenching and consequently provides a measure of Rifabutin’s location within the membrane. The extent of quenching was determined by the Stern–Volmer constant (*K*_SV_), and the efficiency of the quenching of the probes was determined by the bimolecular quenching constant (*K*_q_).^24^ Higher values represent higher quenching and higher proximity of the drug to a given probe.

**Figure 3:**
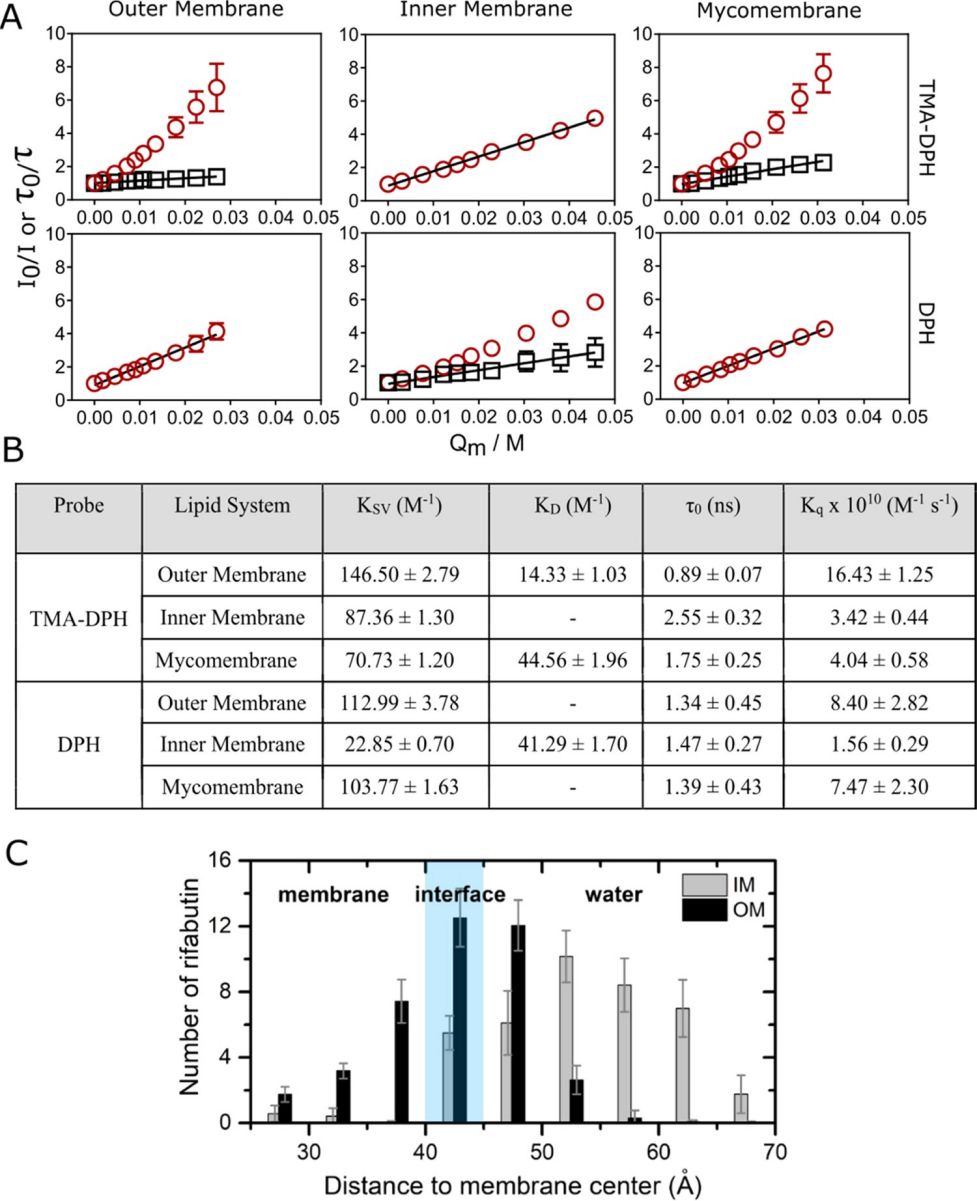
(A) Fluorescence probe quenching to determine the location of Rifabutin within each lipid bilayer. Static quenching  (red circle) and dynamic quenching  (black square) of DPH and TMA–DPH probes were measured against increasing concentrations of Rifabutin (0–30 μM) in each *Msm* membrane system. (B) Stern–Volmer quenching constant (*K*_SV_), dynamic quenching constant (*K*_D_), and bimolecular quenching constant (*K*_q_) were derived from the static and lifetime fluorescence plots to determine the extent of quenching by, and the accessibility of, Rifabutin to DPH/TMA–DPH fluorescence probes embedded in different *Msm* membrane systems, respectively. (C) Number of Rifabutin molecules in the regions at different *z*-direction distances from the membrane center.

In the outer membrane, Rifabutin was found to be located both in the head group/interfacial and hydrophobic acyl chain regions (Figure 3A), with higher quenching efficiency in the head group region. However, the drug was exclusively localized in the interfacial head region in the inner membrane. This may be due to the higher proportion of saturated acyl chains in the inner membrane lipids^4^ that prevent the localization of Rifabutin within the hydrophobic bilayer region as illustrated by modeling as well (Movies S1 and S2). In the mycomembrane, Rifabutin showed similar behavior to that in the outer membrane but higher quenching within the hydrophobic acyl tail region. This indicates that the presence of LAM and PAL in mycomembrane PAL enhances the immersion of Rifabutin, resulting in increased quenching constants in the DPH region.

The depth of immersion of Rifabutin, i.e., the number of Rifabutin molecules in the regions at different *z*-direction distances from the membrane center, also revealed a uniform distribution in the outer membrane, while there was interfacial preferential localization in the inner membrane (Figure 3B). To gain further insights, we used molecular simulations to investigate the lipid–Rifabutin interaction landscape to reveal which lipids in each membrane fraction showed preferential interactions with Rifabutin (Table S5). We found that Rifabutin interacts preferentially with LAM, TDM, and SL-1 and shows no interactions with MA in the mycomembrane. By comparison, in the inner membrane, Rifabutin preferentially interacts with cardiolipin and Ac2PIMs and not PG or DAGs. Exclusive contacts of Rifabutin with LAM in the mycomembrane may result in shielding of the drug from the quencher due to the bulky polar sugar appendages of LAM. As the polar sugar branches of LAM are the defining head group feature of this class of *Mtb* lipids, the shielding of Rifabutin is expected to be maximum in the head group/interfacial region, as experimentally observed from the TMA–DPH quenching data.

Notably, the bilayer positions of these probes have been established in simpler lipid bilayers, and their quantitative positions within mycobacterial lipid membranes (as in this work) are not known. However, based on our previous work on the correlation between the probe’s lifetime and hydrophobic/hydrophilic environment,^4,7^ a deeper penetration of DPH (compared with TMA–DPH) within all mycobacterial membranes is expected. Nonetheless, caution would be exercised with this data, and further work would be required to accurately determine the position of these probes in such complex membrane systems.

Taken together, Rifabutin is localized at distinct bilayer positions within the different *Msm* membrane layers due to specific interactions with chemically and structurally diverse lipids constituting the inner and outer/mycomembrane. In conjunction with distinct partitioning behavior, the data suggests, in the context of whole bacteria, that a drug gradient could exist within the *Msm* membrane cell envelope layers, fine-tuning the intracellular accumulation of drugs and hence resistance. In fact, drug resistance to Rifampicin has been shown to be accompanied by remodeled surface lipid composition.^25,26^

### Rifabutin Alters the Fluidity, Order, and Hydration of Mycobacterial Membrane Layers at High Temperatures

**Figure 4:**
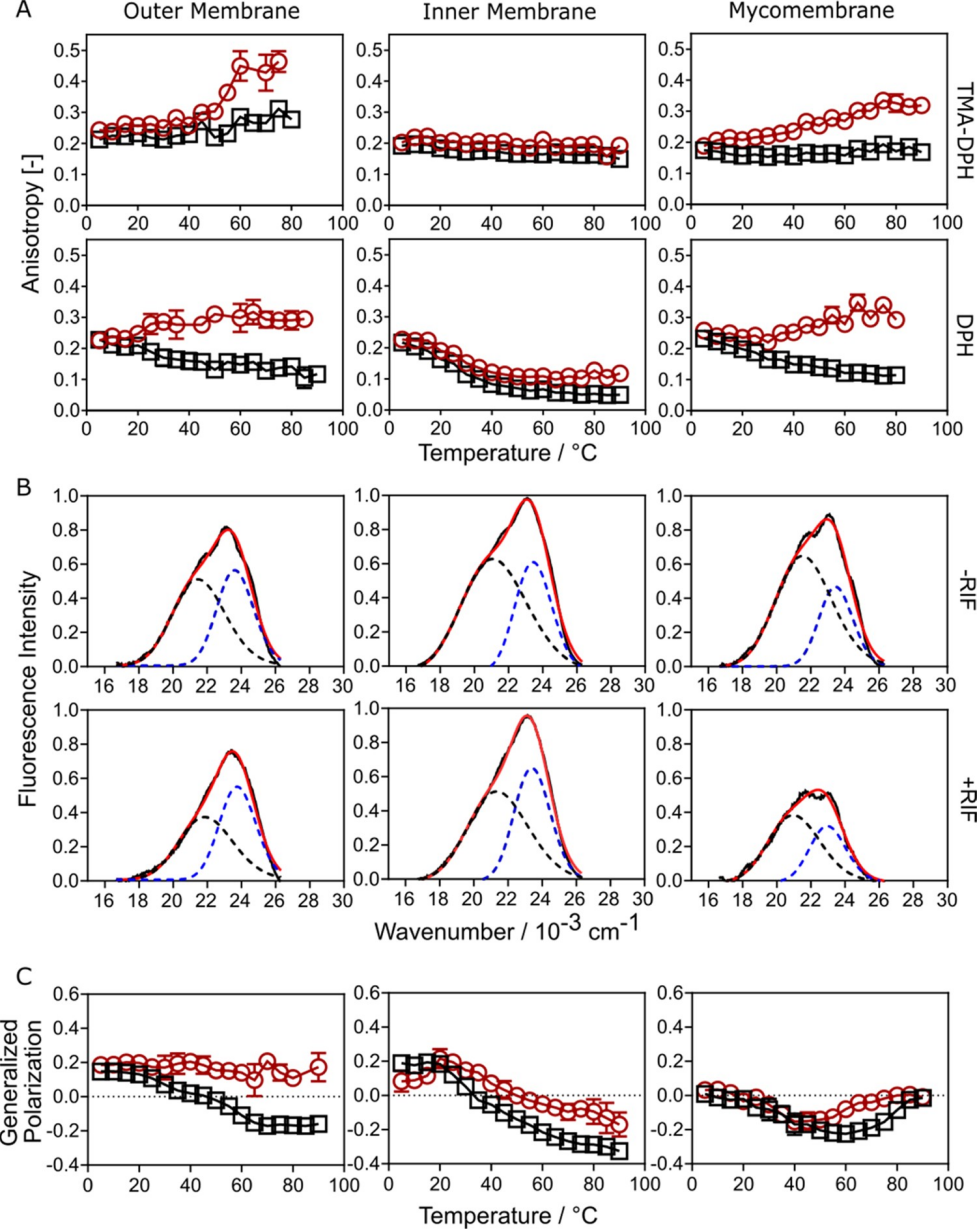
(A) Mycobacterial membrane fluidity perturbations over temperature in the absence (black square) and presence (red circle) of 10 mol % Rifabutin indicated by fluorescence probes TMA–DPH and DPH. (B) Head-group modulations in the presence of 10 mol % Rifabutin tracked with the help of water-sensitive fluorescence probe Laurdan in the indicated *Msm* systems. Laurdan deconvolution of the normalized and baseline-corrected fluorescence intensity curves at 25 °C (—) into charge-transfer states (---) and solvent-relaxed state (blue dashed curves) by the log-normal (LN) deconvolution method (red curves). (C) Generalized polarization (GP) of the lipid systems across various temperatures in the absence (black square) and presence (red circle) of 10 mol % Rifabutin.

To analyze and quantify membrane fluidity (inversely related to membrane microviscosity), steady-state fluorescence anisotropy of DPH and TMA–DPH were used. At ambient temperature, Rifabutin did not affect the fluidity of the *Msm* membranes, both in the head group/interfacial and hydrophobic acyl chain regions (Figure 4A). No change in the lipid tail order parameter (*S*_CD_) in the presence of Rifabutin also supports no major influence on the acyl chain conformation and order/fluidity in the acyl chain region in *Msm* membranes at ambient conditions (Table S2), suggesting weak or transient binding/interactions. This was verified by observing very fast kinetics of Rifabutin binding with, and dissociating from, the *Msm* membranes using surface plasmon resonance (SPR) (Figure S3), indicating that these interactions are relatively transient with no substantial remodeling of membranes. At a physiologically relevant temperature (37 °C), Rifabutin increased the membrane microviscosity (decreased fluidity) within the outer membrane but only in the hydrophobic acyl chain region (DPH). However, Rifabutin caused a decrease in the fluidity of the mycomembrane, in both the head group/interfacial and hydrophobic acyl chain regions (TMA–PDH and DPH, respectively). This indicates that Rifabutin interactions with *Msm* membranes alter membrane fluidity possibly via modulation of membrane lateral organization and leveraging interactions with lipids in the head group/interfacial and acyl chain regions. At higher temperatures (above 50 °C), fluidity in the lipid head group and acyl chain regions for both the outer membrane and mycomembrane decreased in the presence of Rifabutin (Figure 4A and Table S6). This could be due to the temperature-induced ionization of the drug, which could foster electrostatic interactions and hence induce tight packing.^27^ Furthermore, the bulky and rigid Rifabutin structure has the potential to increase membrane packing within the fluid disordered membrane phases inherent to the *Msm* outer membrane and mycomembrane at higher temperatures.^4^ Moreover, interactions of the hydrophobic naphthol residue of Rifabutin with MA and PDIM chains through van der Waals interactions may also lead to efficient packing, causing decreased fluidity.^28^ The increase in microviscosity in the head group/interfacial region in the presence of Rifabutin at these temperatures was attenuated in the mycomembrane compared to its outer leaflet (i.e., the outer membrane), suggesting that the bulky polar head group of LAM mitigates electrostatic interactions of Rifabutin with lipid head groups by shielding the polar head groups, also supported by lipid contact analysis results listed in Table S5.

Next, we evaluated the membrane hydration/order of the lipid bilayers using Laurdan generalized polarization (GP). Laurdan is a solvatochromic dye that enables its fluorescence spectra to be deconvoluted into states with high (longer wavelength) and low hydration (short wavelength) within membrane bilayers.^29^ Similar to fluidity, Rifabutin induced ordering at high temperatures (Figures 4B and S4), probably due to electrostatic interaction of Rifabutin with lipid head groups in the outer membrane and mycomembrane, leading to a rigidified water network in the head group/interfacial region. Moreover, due to Rifabutin’s localization within the hydrophobic acyl chain region in the outer membrane, the displacement of water further increases the order of the outer membrane compared to that of the inner membrane.

Our data points to a gradient in the degree of Rifabutin-induced changes in the fluidity/order across the spatially resolved mycobacterial cell envelope layers in the order outer membrane > mycomembrane > inner membrane. Also, the lipid composition unique to different layers^4^ further fine-tunes the balance between Rifabutin’s hydrophobic, polar and electrostatic interactions with the *Msm* membranes. Thus, active remodeling of the mycobacterial cell envelope in response to various factors represents an attractive strategy for the bacteria to modulate its membrane interactions with various external agents like drugs or from the host, fostering its survival and pathogenesis. This is further supported by findings from Howard et al. that revealed mycobacteria carrying rifamycin resistance to display modifications in their cell surface lipid profile that favor pathogen survival.^25^

### Rifabutin Does Not Induce Macroscopic Perturbation of *Msm* Membranes

**Figure 5:**
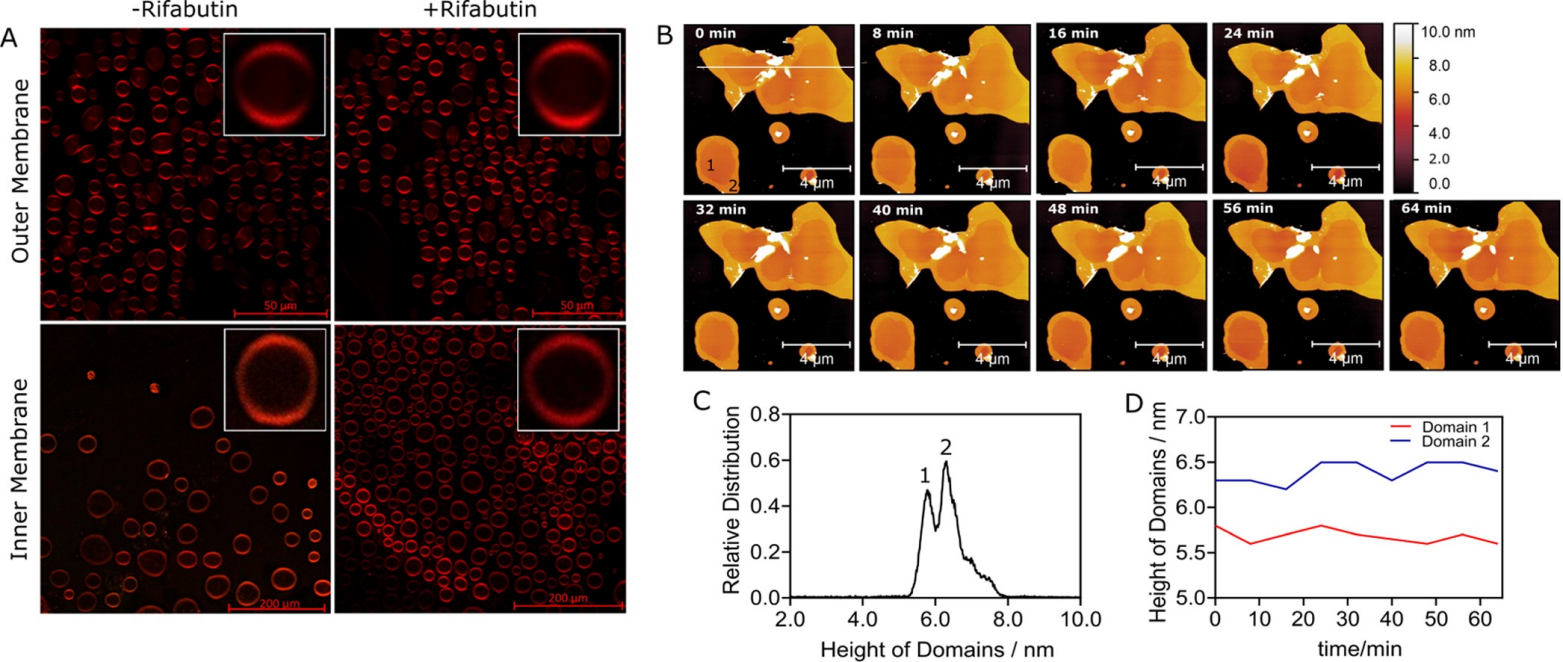
(A) Confocal imaging of the *N*-Rh-DHPE-labeled *Msm* systems. Both ordered (devoid of *N*-Rh-DHPE signal) and disordered regions (with *N*-Rh-DHPE signal) in the membranes are visualized. Rifabutin addition did not alter the abundance or distribution of disordered domains in three replicate studies. (B) Topography of inner membrane observed as solid-supported bilayers (SLBs) with atomic force microscopy. (C) Inner membranes displaying at least two significant lipid domains of ∼5.8 nm (domain 1) and ∼6.3 nm (domain 2) in height. (D) Temporal changes in the height of lipid domains after the addition of 25 μM Rifabutin.

Here, we investigated the lipid membrane domain organization in the presence of Rifabutin using giant unilamellar vesicles (GUVs) with confocal microscopy and solid-supported bilayers (SLBs) with atomic force microscopy (AFM). Confocal microscopy revealed that both the inner and outer membranes displayed lateral phase segregation with at least two macroscopic membrane domains (Figure 5A): disordered regions harboring *n*-(lissamine rhodamine B sulfonyl)-1,2-dihexadecanoyl-*sn*-glycero-3-phosphoethanolamine (*N*-Rh-DHPE) that partitions specifically into the liquid-disordered phase of a membrane and ordered regions devoid of the fluorophore. In situ addition of Rifabutin did not induce any major perturbation of the lateral phase organization in *Msm* membranes (Figure 5A and Table S7). Next, using AFM in SLBs, topographical analysis of the *Msm* inner membrane confirmed the presence of at least two lipid phases (Figure 5B) differing in height by approximately 1–2 nm, and the overall bilayer height/thickness (Figure 5C) was 5.5–6.5 nm, in accordance with previous studies.^4,18,19^ In the presence of Rifabutin did not change the overall thickness of the domains (Figures 5D and S5), also supported by simulations.

**Figure 6:**
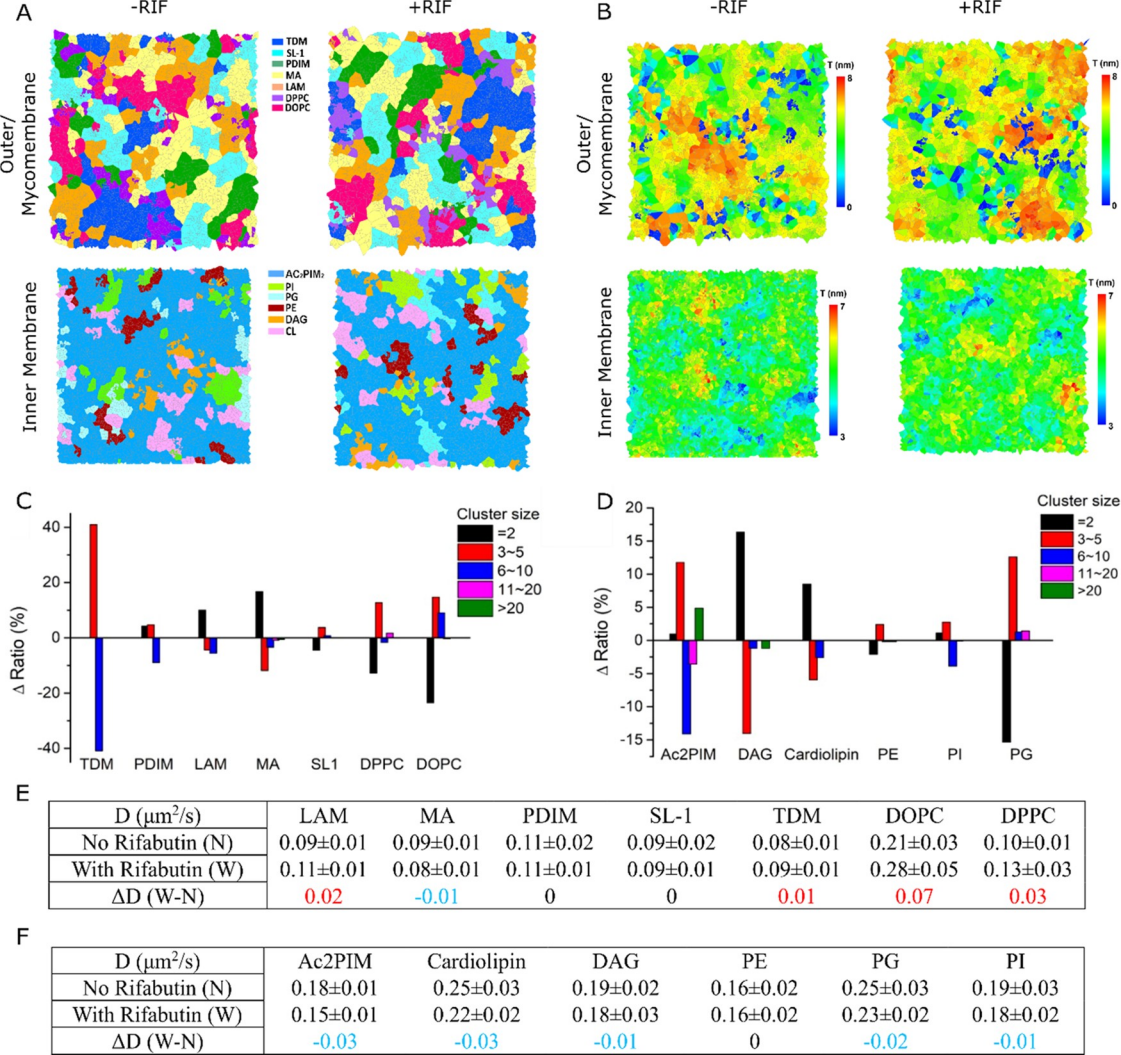
Molecular dynamics (MD) simulations of the mycomembrane and inner membrane to determine the membrane properties. (A) Domain distribution and (B) height heterogeneity in the absence and presence of Rifabutin to study the influence of Rifabutin on the membranes. The Δ ratio of different size clusters of lipids in (C) mycomembrane and (D) inner membrane. The Δ ratio values are calculated by the ratio changes influenced by Rifabutin. Diffusion rate of the lipids in (E) outer membrane/mycomembrane and (F) inner membrane with or without Rifabutin molecules calculated from the mean-square displacement (MSD) of these molecules.

This prompted us to investigate whether the sub-microscopic membrane lateral organization of *Msm* membranes was altered by the drug. Our simulations revealed that *Msm* membranes exhibit distinct lipid-domain nanoclustering (lipid clustering maps, Figure 6A),^30^ with the presence of lipid domains of variable heights (Figure 6B). Upon the addition of Rifabutin, the lateral nanodomain clustering was redistributed. Specifically, lipid-domain cluster size distribution within *Msm* membranes in the presence of the drug was modulated (Figure 6C,D). For the mycomembrane, bigger clusters of TDM, MA, LAM, and PDIM (with 6–10 lipids) decreased and smaller-sized clusters (2–5 lipids) increased in the presence of Rifabutin. For the inner membrane lipids, bigger clusters of AC_2_PIM (>20 lipids), PG, and PI increased, indicating that the drug may induce clustering of AC_2_PIM lipids. These results likely signify changes in the cell envelope membrane organization in response to lipid structure-dependent interactions with Rifabutin. These are also indicated by the various lipid–Rifabutin contact numbers against various lipids within the inner membrane and mycomembrane (Table S5). Various studies on model and cellular membranes have revealed that chemical distinct antiraft acting drugs can both reduce and induce lipid clustering to remodel the functional protein landscape within lipid clusters and the surrounding membranes, affecting the membrane organizational integrity associated with the pharmacological activity of these drugs.^31^ Of note, increasing the time of simulations did not change the conclusions of this work (Figures S6 and S7 and Tables S8–S11), indicating that our membrane model reached equilibration, despite having complex lipid structures. Our modeling data implies that Rifabutin alters the lipid clustering distribution distinctly within the two layers and could be attributed to altered inter/intralipid–lipid interactions that govern clustering but need experimental verification nonetheless.

However, there were no changes in the diffusion rate (*D*) of constituent lipids within *Msm* membranes (Figure 6E), suggesting no substantial membrane reorganization.

## Conclusions

Mycobacterial species exhibit robust self-defense against many antibiotics and host molecules, and this is mainly governed by the pathogen’s complex cell envelope lipid membrane architecture. Using membrane biophysics and simulations, we show that the distinct mycobacterial cell envelope membrane layers differ in their interaction with antibiotic Rifabutin. Rifabutin partitions the least within the outer membrane/mycomembrane but distributes itself uniformly within the membrane head group/interfacial and hydrophobic acyl chain regions, possibly due to favorable interactions with the constituent lipids. In contrast, Rifabutin showed the highest partitioning in the inner membrane but preferred to situate and/or aggregate in the interfacial lipid head group region only. This could be due to attenuated contacts with the inner membrane lipids, limiting its penetration to the deeper/bilayer regions and restricting the drug penetration only in the interfacial region. Also, a nonspecific mechanism driving enhanced penetration of Rifabutin into the inner membrane interfacial region is proposed, but this needs further verification. The outer membrane/mycomembrane seems to act as a barrier against optimal drug penetration due to limited drug partitioning. Thus, membrane-active agents capable of selectively disrupting the outer membrane/mycomembrane structure could enhance drug uptake by mitigating the limited partitioning of Rifabutin and eventually increase its intracellular concentration; Rifabutin already demonstrated enhanced partitioning in the inner membrane but limited penetration within the deep hydrophobic region. This approach could be explored as combination therapy in TB^32^ and is also likely to counteract the origin of resistance associated with limited drug permeability (causing low intracellular concentrations). The drug did not induce any major perturbation of the membrane structure, organization, order, and fluidity in either membrane layer, but sub-microscopic reorganization of lipid nanodomain clustering is proposed. Altered lipid clustering is expected to modulate the protein-localizing function of membrane domains, thereby affecting the protein–membrane structural and functional integrity and impacting the physiological functions of bacteria.

It is emphasized that these findings are derived from simplified protein-free membrane mimics of the *Msm* cell envelope; thus, the role of membrane proteins in regulating drug–membrane interactions and hence the complete biological functions of mycobacterial membranes remain to be investigated. Furthermore, observations made in *Msm* would require verification in the homologous bacteria for a complete understanding of the membrane properties that impact drug passage and retention within pathogenic and clinical species.

This work has a number of implications in regulating mycobacterial physiology and pathology. First, limited drug partitioning within the outer membrane suggests that this layer regulates the selective entry of molecules to reach the inner membrane. Second, from the quenching and membrane dynamics studies, it is clear that the inner membrane behaves like a sink that prevents the molecules’ passage across its bilayer (by accumulating the drug in the interfacial region). Thus, as shown for many bacteria, environmental factors such as host and drug exposure may induce changes in the mycobacterial lipidome that affects membrane composition^25,33^ and their interaction with exogenous agents. Hence, a detailed understanding of drug–membrane interactions in a lipid composition selective manner is thus expected to reveal novel insights into developing membrane-inspired antitubercular therapeutic approaches with significantly reduced drug resistance.

## Materials and Methods

### Materials

*M. smegmatis* mc^2^ 155 (ATCC # 700084) was a humble gift from Dr. S. Chopra’s lab (CDRI, India). Dehydrated culture media of Middlebrook 7H9 broth was purchased from BD Difco. BSA fraction V was procured from MP Biochemicals, Southern California. Rifabutin and lipid probes diphenylhexatriene (DPH) and trimethylamino-diphenylhexatriene (TMA–DPH) were purchased from Cayman Chemicals. The probe *n*-(lissamine rhodamine B sulfonyl)-1,2-dihexadecanoyl-*sn*-glycero-3-phosphoethanolamine (*N*-Rh-DHPE) was purchased from Avanti Polar Lipids. Chloroform of spectroscopic grade was purchased from Spectrochem. Probe Laurdan and other chemicals (dextrose, catalase, D_2_O, Tris, *N*-(2-hydroxyethyl)piperazine-*N*′-ethanesulfonic acid (HEPES), NaCl, MgCl_2_, and methanol of spectroscopic grade) were purchased from Sigma-Aldrich. Tween 80 and anhydrous glycerol were bought from Merck and EMPARTA, respectively. All of the products were used without further purification. The water used for aqueous buffer solutions was from a Millipore water purification system.

### Bacterial Cell Culture

*M. smegmatis* mc^2^ 155 (*Msm*) was grown in Middlebrook 7H9 medium supplemented with 10% (v/v) in-house prepared albumin–dextrose–catalase (ADC), 0.1% Tween 80, and 0.5% glycerol and cultured at 37 °C under 120 rpm shaking conditions. Cells to be harvested at early carbon-limited stationary phase were grown separately in Tween-free medium until reaching OD_600_ 3.0.^34−36^ Cells were washed twice with phosphate-buffered saline (pH 7.4) before lipid extraction.

### Lipid Extraction

Lipids were extracted by previously reported methods.^3,4^ Briefly, noncovalent outer membrane lipids were selectively extracted using 1 mL of reverse micellar solution (RMS; 10 mM sulfosuccinic acid 1,4-bis (2-ethylhexyl) ester sodium salt (AOT) in heptane) for every 10 mg of dry weight of cells. For the inner membrane lipid extraction, the RMS-treated cells were washed twice with distilled water and then extracted with 3 mL of chloroform/methanol/water (2:1:0.1) for every 10 mg of dry mass. Both extraction steps were carried out in monophasic solutions four times, with the first extraction spanning overnight and the rest spanning 30 min each. The extracts were vacuum-dried to obtain respective lipid fractions.

The outer membrane lipid fraction was further purified from its AOT mixture using column chromatography of 100–200 silica mesh with a mobile phase of gradient methanol in chloroform (up to 8%).^4^ Almost all lipids, with no AOT, were eluted at up to 8% methanol, which was confirmed from the fractions run on thin-layer chromatography (TLC) plates developed by 1% anthrone spray.

For the extraction of peptidoglycan-associated lipids (PALs), 10 mg of delipidated cells (cells after the removal of noncovalently attached lipids) was washed and then treated with 10 mg of lysozyme in 1 mL of 10 mM sodium phosphate buffer (pH 7.5) under 150 rpm for 2 h at 37 °C. These lysozyme-treated cells were re-extracted four times with chloroform/methanol/water (2:1:0.1) in the same manner as done for the inner membrane lipids. Lipoarabinomannans (LAMs)/lipomannans (LMs) were also extracted from delipidated cells. The cells were washed and broken by sonication followed by refluxing every 10 mg of cells thrice in 20 mL of 50% aqueous ethanol for 4 h. The extract was obtained by centrifugation at 3500*g* after each reflux. The extract was dried and resolubilized in phosphate-buffered saline (PBS) to 0.5 mg/mL. An equal volume of monophasic PBS-saturated phenol was added, and the mixture was kept in a water bath at 75 °C for 20 min, followed by ice-bath for 20 min and finally left at room temperature (RT) for 20 min. The aqueous layer was collected by centrifuging at 27 000*g*, and the phenol layer was back-extracted twice using equal volumes of PBS. LAM/LM was obtained from this combined aqueous phase by dialyzing against water (molecular weight cutoff of 3500 Da). LAM and PAL were added to the outer membrane lipid fraction at the concentrations found in physiologically relevant conditions^3^ to generate the mycomembrane (69.64:1.56:28.8 mol %; outer membrane lipids/LAMs/PALs) using average molecular weights calculated previously.^4^

### Analysis of Lipids

The lipids extracted were analyzed using thin-layer chromatography (TLC) plates with different mobile phases and development sprays depending upon the lipids of interest and polarity.^37^ LAM was detected using sodium dodecyl sulfate-polyacrylamide gel electrophoresis (SDS-PAGE), and its molecular size was compared with a standard size ladder.

### Preparation of Liposomes

Liposomal suspensions of different extracted lipids were prepared by a hydration method. Briefly, the lipid fractions were diluted in chloroform in the presence of lipid probes *N*-Rh-DHPE (0.1 mol %)/Laurdan (0.5 mol %)/TMA–DPH (1 mol %)/DPH (0.5 mol %) to obtain a final lipid concentration of 0.5 mg/mL unless stated otherwise. The lipid film was prepared by drying chloroform under a stream of nitrogen gas followed by drying under reduced pressure conditions overnight. For UV–vis and fluorescence spectroscopy assays, the film was hydrated with 0.22 μm filtered aqueous buffer (20 mM Tris, 5 mM MgCl_2_, pH 7.4) at RT and was sonicated for 15 min, followed by five freeze–thaw cycles to generate large unilamellar vesicles (LUVs).^38^ For the drug–lipid membrane studies, Rifabutin was added in 10:1 lipid/drug molar ratio^5^ unless specified otherwise and incubated for a period of 1 h in the dark at 37 °C. For SPR and AFM, the lipid film was hydrated with 0.22 μm filtered aqueous buffer (10 mM HEPES, 150 mM NaCl, pH 7.2) to a final concentration of 1 mg/mL with constant vortexing at RT, incubated at 37 °C for 1 h at 180 rpm/min, and then sonicated for approximately 30 min until fully clear. Homogeneity in the size was obtained by passing the clear liposome solution through a 100 nm polycarbonate membrane 31 times.^39^

### Molar Volume (*V*_m_) Determination

*V*_m_ of lipid mixtures was determined by a neutral buoyancy method based on earlier studies.^40,41^ Different ratios of D_2_O in H_2_O were used to hydrate lipid films to generate the liposomes. The mass fraction of D_2_O (φ_D_2_O_) in the solvent was determined by eq 1.
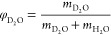
1where *m*_D_2_O_ and *m*_H_2_O_ represent the masses of D_2_O and H_2_O, respectively.

The inverse density of the final solvent (*V*_sol_) was obtained by eq 2.
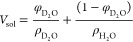
2where ρ_D_2_O_ and ρ_H_2_O_ represent the densities of D_2_O and H_2_O, respectively, at 25 °C.

Assuming that the interlamellar solvent and the bulk solvent are the same after liposome preparation and following the principle that LUVs sink when suspended in a solution with lower density than the lipids and floats in a higher-density solution, the specific volume (*V*_S_) of the lipids will fall in the range between solutions in which they float and sink.

Liposomal suspensions prepared from a broader range of *V*_sol_ (*V*_A_ and *V*_B_) were centrifuged at 19 800*g* for 30 min at 25 °C and checked visually whether the liposomes had sunk or were afloat. The range was narrowed down to *V*_A_ – *V*_B_ = 0.01 mL/g (where the liposome sinks in *V*_A_ and the liposome floats in *V*_B_), thereby determining the *V*_S_ within ±0.005 mL/g. The obtained *V*_S_ is the average of the final *V*_A_ and *V*_B_.

The procedure was validated by deriving the specific volume of dipalmitoylphosphatidylcholine (DPPC) to be 0.941 mL/g, where the reported value stands 0.94 mL/g at 25 °C.

From the theory of the neutral buoyancy method, eq 3 was used
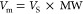
3where MW represents the equivalent molecular weight of the lipid mixtures.

### Determination of Partition Coefficient (*K*_p_)

The partition coefficient of Rifabutin between the lipid membranes and the aqueous buffer was determined using the derivative UV–visible spectrometry technique.^5,42−44^ Absorption of UV light by different concentrations of liposomes (0–1000 μM) with and without the addition of Rifabutin (25 μM) was measured at RT using a Thermo Scientific Evolution 201/220 UV–visible spectrophotometer. The intensities were then processed to obtain the third derivative with respect to the wavelengths using the nprot *K*_p_ calculator.^14^ The third derivative intensities ranging from 284 to 297 nm were considered for each lipid system to calculate the *K*_p_ by fitting the experimental data to eq 4 by a nonlinear regression method using Origin 2019b.
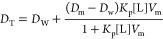
4where *D* represents the third derivative intensity  obtained from the absorbance of total Rifabutin concentration (*D*_T_), Rifabutin distributed in lipid membrane phase (*D*_m_), and Rifabutin distributed in the aqueous phase (*D*_w_); [L] is the molar lipid concentration; and *V*_m_ is the molar volume derived earlier for each lipid mixture.

### Drug Location within Lipid Membranes Using Fluorescence Quenching

The extent of quenching experienced by the fluorescent probes—DPH (ex, 357 nm; em, 430 nm), positioned near the acyl chains, and TMA–DPH (ex: 355 nm, em: 430 nm), positioned closer to the polar head groups—in the presence of Rifabutin indicates the location of the drug within membrane bilayers.^45−49^

Rifabutin at a concentration range of 0–30 μM was added to DPH/TMA–DPH-tagged liposomal solutions, followed by incubation in the dark for 20 min. The fluorescence intensities emitted by the probes were captured at 37 ± 0.1 °C using a Varian Cary Eclipse fluorescence spectrophotometer. For lifetime quenching studies, a 375 nm laser excitation source was used. The fluorescence lifetime decay was measured on a picosecond pulsed diode laser-based time-correlated single-photon counting (TCSPC) system from IBH, U.K. with a repetition rate of 1 MHz and full width at half-maximum (FWHM) of the instrument response function (IRF) of ∼270 ps, and a photomultiplier tube (PMT) was used as the detector. The fluorescence lifetime decay spectra were fitted using v6.2 IBH DAS software through an iterative reconvolution method with the χ^2^ value ranging from 1 to 1.2. The lifetime contributions after fitting and the intensities at 430 nm obtained from spectrofluorometer are utilized for determining the patterns of quenching with the aid of either the Stern–Volmer or modified Stern–Volmer equation (eq 5) using GraphPad Prism8.

Considering the *K*_p_ of Rifabutin for each membrane system, only a portion of the drug gets partitioned into them. Hence, to derive the linearity of the quenching of the probes, the effective concentration of the drug (*Q*_m_) contributing to the quenching was calculated using eq 5.
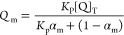
5where [Q]_T_ represents the total Rifabutin concentration and α_m_ represents the volume fraction of the membrane phase.
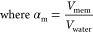
6where *V*_mem_ and *V*_water_ represent the volumes of the membrane and water phase, respectively. The fluorescence intensity of the probes could be quenched due to various molecular interactions, and the probability of both static and dynamic quenching was considered. The Stern–Volmer equation noted below is used to determine the ability of the drug to quench the probes.
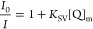
7where *I*_0_ and *I* are the steady-state fluorescence intensities with and without the drug and *K*_SV_ is the Stern–Volmer constant. The line thus derived would be linear with the goodness of fit (*R*^2^) >0.990. The absence of linearity would mark the involvement of the dynamic component in quenching, which can be calculated using the modified Stern–Volmer equation given below
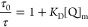
8
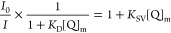
9where τ_0_ and τ are the lifetimes with and without the drug and *K*_D_ is the dynamic quenching constant. The resulting line with *K*_SV_ as the slope would be linear with a goodness of fit (*R*^2^) >0.990. *K*_SV_ indicates the static quenching effect imparted by the drug on the probes. It can be further utilized to determine the bimolecular quenching constant (*K*_q_, eq 10), which would represent the effect of both static and dynamic quenching.
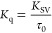
10

### Fluorescence Anisotropy

Membrane fluidity at various bilayer depths was measured by means of fluorescence anisotropy that provides information on membrane microviscosity (inversely related to fluidity). The changes in the local environment of the fluorophores within a membrane system can be marked by monitoring the orientation and rotational correlation time, which would be indicated by fluorescence anisotropy.^50,51^ Fluorescence anisotropy of DPH/TMA–DPH-labeled lipid membranes, in the presence and absence of the drug, was measured using a temperature-controlled Varian Cary Eclipse fluorescence spectrophotometer attached with a polarizer (Varian Cary Eclipse Manual Polarizer), considering the range of temperature from 5 to 90 °C giving a period of 3 min for equilibration with an accuracy of ±0.1 °C. The samples were excited with vertically and horizontally polarized lights, and respective polarized emission intensities were recorded. The degree of fluorescence steady-state anisotropy (*r*) was calculated from the following equation.^52−54^
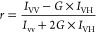
11where *I*_VV_ and *I*_VH_ are the parallel and perpendicular emission intensities of the vertically polarized excitation beams, respectively,  is the correction factor to determine the sensitivity of the instrument (*G* should be ∼1), and *I*_HV_ and *I*_HH_ are the parallel and perpendicular emission intensities of the horizontally polarized excitation beams, respectively.

### Laurdan Generalized Polarization (GP) Spectroscopy

Membrane packing-sensitive fluorescence probe Laurdan (ex, 350 nm; em, 440/490 nm) when exposed to water molecules experiences solvent relaxation, resulting in a red shift.^55^ The fluorescence intensities of Laurdan incorporated within lipid vesicles, with and without Rifabutin, were recorded using a temperature-controlled Varian Cary Eclipse fluorescence spectrophotometer, considering the range of temperature from 5 to 90 °C, giving a period of 3 min for equilibration with an accuracy of ±0.1 °C.

The use of the generic formula of  (where *I* is the intensity at specified wavelengths) for LUVs containing complex lipid mixtures might not be a good indication to point out the changes imparted to the lipid head groups. Hence, GP was obtained from the spectra after log-normal spectral decomposition.^29,56^ Each spectrum of Laurdan was treated as a superposition of two log-normal (LN) functions—one of each representing the two excited states of Laurdan: the nonrelaxed (blue channel) and the relaxed (green channel) states.

The raw data at each temperature was converted to fit in the wavenumber format—with wavenumber on the *x*-axis and intensity corresponding to the wavenumber (*I* = *I*_λ_ × λ^2^, where *I*_λ_ is the intensity in wavelength scale and λ is the wavelength) on the *y*-axis. The spectra thus obtained are normalized, baseline-corrected, and then subjected log-normal deconvolution using Origin 2019b to two peaks (charge-transfer state, solvent relaxation state) or three peaks (also included locally excited state), and the two intensities corresponding to charge transfer (*I*_B_ for the blue channel) and solvent relaxation (*I*_G_ for the green channel) were picked. The GP curve is plotted using the following equation.
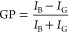
12

### Surface Plasmon Resonance (SPR) Spectroscopy

SPR exploits the change in the resonance, generated by the wavefield of plasmons, with every change in the refractive index on the metal surface.^57^ The liposome solution was diluted to a 0.28 mg/mL solution containing 2 mM CaCl_2_. Rifabutin stock was prepared in dimethyl sulfoxide (DMSO), and its subsequent drug dilutions ranging from 1 to 60 μM concentrations were prepared maintaining the final DMSO concentrations in the samples as 0.2%. All samples and buffers were degassed before loading the experiment.

The L1 sensor chip was primed with Milli-Q water and then with HEPES-buffered saline (HBS) buffer for 6 min each. The surface was then washed with the regeneration solution—injections of 40 mM 3-cholamidopropyl dimethylammonio 1-propanesulfonate (CHAPS) at a flow rate of 5 μL/min followed by 5 μL of 2-propanol/50 mM sodium hydroxide (2:3, v/v) twice for 60 min each. LUVs were then injected to the sensor chip at a flow rate of 2 μL/min for 3000 s for liposome capture. To remove the Ca^2+^ ions and any multilamellar structures from the lipid surface, 30 mM ethylenediaminetetraacetic acid (EDTA) in HBS buffer was injected at a flow rate of 20 μL/min, which resulted in a stable baseline corresponding to the supported lipid bilayer. Drug dilution was injected over the lipid surface at a flow rate of 30 μL/min for 240 s to avoid any limitation by mass transfer. Upon completion of the injection, the buffer flow was continued to allow a dissociation time of 600 s. All binding experiments were carried out at 25 °C. After each liposome–drug interaction at each drug concentration, the surface was regenerated using the above-indicated regenerating solutions. The sensorgram obtained was blank-subtracted to eliminate the effect of DMSO and study the interaction of Rifabutin with the lipid membranes.

### Atomic Force Microscopy

Atomic force microscopy was used to examine the influence of Rifabutin on the topography of mycobacterial membranes.^58^ The liposomes were prepared as in the SPR experiment and diluted to 0.5 mg/mL with the addition of 4 mM CaCl_2_. To freshly peeled mica, 200 μL of 0.5 mg/mL liposomal solution was added and kept at RT overnight in a clean humid environment to form SLBs. The unbound lipids were washed out, and fresh HBS buffer was added. The topography of the SLBs was characterized using PeakForce Mapping in Fluid in Nanomechanical Mapping mode on a FastScan Bio AFM (Bruker AXS, CA) controlled with Nanoscope 9.7 software. A triangular ScanAsyst-Fluid+ probe (Bruker, CA) with a nominal tip of 2 nm and a nominal spring constant of 0.7 N/m was used, and imaging was performed in the fluid condition. The SLBs formed on the mica were scanned with a droplet method, wherein the probe loaded onto the scanner was prewet with 30 μL of HBS followed by engaging the sample. The force setpoint was manually maintained in between 600 and 900 pN with the feedback gain automatically adjusted by software. The amplitude and frequency of peak force were set at 70–80 nm and 2 kHz, respectively. The topographic images were analyzed with NanoScope Analysis software and processed using Gwyddion 2.56 software.

### Formation of Giant Unilamellar Vesicles (GUVs) and Fluorescence Microscopy

GUVs were prepared by a temperature-controlled electroformation method.^4^ The size (1–100 μm) of GUVs would aid in visualization under a microscope and therefore to determine the lateral membrane organization when GUVs are labeled with *N*-Rh-DPPE, which partitions into the disordered regions of the lipid membrane.^38,59^ About 60 μL of 4 mg/mL lipid mixtures containing *N*-Rh-DPPE (ex, 561 nm; em, 592 nm) was spin-coated at 800 rpm on to the optically transparent and electrically conductive indium tin oxide (ITO)-coated glass coverslip (SPI Supplies, West Chester) and then dried at reduced pressure conditions. The coverslip was placed in a custom-made electroformation cell and then 0.22 μm filtered Milli-Q was added to the cell. The lipid film was hydrated at 65 °C before applying the low-frequency alternating current field (2 V and 10 Hz) to the ITO electrodes using a function generator (Tektronix AFG 1022 Instruments) for 3 h, and then, the cell was gradually cooled down to RT at a constant rate of 1 °C/min.

GUVs were acquired using a 561 nm diode-pumped solid-state (DPSS) laser source using a laser scanning confocal microscope (LSM 780, Carl Zeiss, Germany) and an Olympus (Tokyo, Japan) plan-apochromat 10×/0.45 M27 air objective at RT. For drug–membrane studies, the Rifabutin solution was added to the electroformation cell slowly from a side port and incubated at 37 °C in the dark for 1 h before imaging as described above. All images collected were analyzed as 8-bit, unsigned images with 1024 × 1024 pixels; *Z*-stack images were captured using a photomultiplier tube (PMT) detector, and the maximum intensity projection (MIP) of the images was obtained whenever required using Zen software (Carl Zeiss). The images obtained were processed using Zen 3.1 (blue edition). The area distribution of the disordered region in 40 GUV/lipid mixture/replicate was measured, and the changes across different membranes were studied.

### MD Simulations

The structure of a single lipid molecule was built and optimized by GaussView.^60^ For lipids DOPC and DPPC, the force field parameters were achieved from Lipid14.^61^ For the other lipids that are not included in Lipid14, the partial charge by fitting the electrostatic potentials through the restrained electrostatic potential (RESP) method was calculated^62^ based on the antechamber module.^63^ The atom types, bonded interaction parameters, and van der Waals interaction parameters were defined by the general Amber force field (GAFF)^64^ and Lipid14 force fields. For each type of lipid, a single molecule was dissolved in water and a short MD simulation was performed to optimize the structure. The lipid bilayer membranes were packed by Packmol software.^65^ Both of the inner membrane and outer membrane/mycomembrane were composed of 800 different lipid molecules, and the ratio of lipid components in the inner membrane was Ac_2_PIM_2_/cardiolipin/DAG/PI/PG/PE = 50:10:10:10:10:10%.^3^ The ratio of lipid components in the outer membrane/mycomembrane was SL-1/TDM/PDIM/LAM/DOPC/DPPC/MA = 10:10:10:10:15:15:30%.^3^ A total of 56 000 and 78 000 TIP3P water molecules were added to build the solvent box for the inner and outer membrane/mycomembrane, respectively. The box sizes for the inner and outer membrane/mycomembrane were 157 × 150 × 122 and 166 × 160 × 155 Å^3^, respectively, and sodium ions were added to neutralize the system. The steepest descent method was performed to minimize the system until the root mean square of energy gradient was <0.0001 kcal/(mol Å) or the maximum iteration steps reached 10 000. The system was then heated to 300 K linearly in the periods of 100 ps in the NVT ensemble with weak harmonic potential (10 kcal/(mol Å)) on the heavy atoms. Subsequently, a 1 ns unrestrained equilibration with a Langevin thermostat^66^ in the NPT ensemble was performed. The bonds involving hydrogen were constrained with the SHAKE algorithm. Then, 500 ns production runs were carried out by CUDA-version Amber16^67^ to equilibrate the membrane systems.

The Rifabutin molecules were randomly inserted into the aqueous solvent layer of the inner and outer membrane/mycomembrane. The initial structure of Rifabutin was built by GaussView.^60^ The partial charges of the atoms of Rifabutin were obtained by the antechamber based on the AM1-BCC calculation. The general Amber force field (GAFF) was utilized to complement other parameters of Rifabutin. The simulation parameters of the membrane with Rifabutin molecules were the same as those for the pure lipid systems. The 1 μs simulation runs were performed for both the membranes with and without Rifabutin, and the trajectories were utilized in the following analysis.

The structure and force field parameters of Rifabutin and lipids are provided in the Supporting Information.

#### Diffusion Analysis

The lipid lateral diffusion in the membrane plane was calculated from the mean-square displacement (MSD) of these molecules
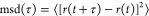
13where ***r***(*t*) is the position of the center of mass (COM) of lipids in the lateral direction at time *t* and τ is the lag time to calculate the displacement of the position in the time step. The lateral diffusion constant (*D*) of a given lipid was calculated based on the 300–500 ns trajectories by fitting the MSD curve
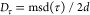
14where *d* is the dimensionality and *d* = 2. The stfc diffusion module in Amber16^67^ was used for calculating the MSD curves and estimating the diffusion constants and their errors.

#### Cluster Size Determination

To calculate the cluster sizes of different lipids, a neighbor connectivity search was performed on each lipid type. The center of mass of any two identical lipids in the neighboring grids with distances shorter than 10.0 Å was considered to be in the same cluster. The DBSCAN algorithm (density-based spatial clustering of applications with noise)^68^ was used to identify the clusters. The average lipid–Rifabutin contact number was calculated by
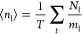
15where *N*_l_ is the total contact number between Rifabutin and lipid l in snapshot *t*, *m*_l_ is the number of lipid l, and *T* is the snapshot number in the last 200 ns simulations. The numbers in Table S2 are equal to 100 × ⟨*n*_l_⟩.

#### Order Parameter Calculation

Lipid tail order parameters (*S*_CD_) were calculated for each lipid tail according to
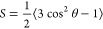
16where θ is the angle between two vectors of the C–H bond of all carbons in a lipid tail and the normal of the bilayer surface. The average order parameter of a particular lipid tail over all of the carbon atoms in the tail was used to estimate the flexibility of the lipid. The cpptraj module of the Amber16 program was applied to analyze the order parameter (*S*_CD_) of the SL-1 bilayer.

## Supporting Information Available

Structures and force fields (ZIP)
Inner membrane side view (Movie S1) (MPG)
Inner membrane top view (Movie S2) (MPG)
Outer membrane side view (Movie S3) (MPG)
Outer membrane top view (Movie S4) (MPG)
Figures and tables related to lipid extraction and identification and SPR, AFM, and modeling data supporting the main conclusions of the work (PDF)

## Supplementary Material

bg2c00010_si_001.zip

bg2c00010_si_002.mpg

bg2c00010_si_003.mpg

bg2c00010_si_004.mpg

bg2c00010_si_005.mpg

## References

[ref1] WHO. Global Tuberculosis Report 2020; World Health Organization: Geneva, 2020. https://www.who.int/publications/i/item/9789240013131.

[ref2] Farhat M. R.; Sixsmith J.; Calderon R.; Hicks N. D.; Fortune S. M.; Murray M. Rifampicin and rifabutin resistance in 1003 *Mycobacterium tuberculosis* clinical isolates. J. Antimicrob. Chemother. 2019, 74, 1477–1483. 10.1093/jac/dkz048. 30793747 PMC6524487

[ref3] Bansal-Mutalik R.; Nikaido H. Mycobacterial outer membrane is a lipid bilayer and the inner membrane is unusually rich in diacyl phosphatidylinositol dimannosides. Proc. Natl. Acad. Sci. U.S.A. 2014, 111, 4958–4963. 10.1073/pnas.1403078111. 24639491 PMC3977252

[ref4] Adhyapak P.; et al. Dynamical Organization of Compositionally Distinct Inner and Outer Membrane Lipids of Mycobacteria. Biophys. J. 2020, 118, 1279–1291. 10.1016/j.bpj.2020.01.027. 32061274 PMC7091463

[ref5] Pinheiro M.; et al. Differential Interactions of Rifabutin with Human and Bacterial Membranes: Implication for Its Therapeutic and Toxic Effects. J. Med. Chem. 2013, 56, 417–426. 10.1021/jm301116j. 23215016

[ref6] Pinheiro M.; et al. The influence of rifabutin on human and bacterial membrane models: implications for its mechanism of action. J. Phys. Chem. B 2013, 117, 6187–6193. 10.1021/jp403073v. 23617457

[ref7] Dadhich R.; et al. Biophysical characterization of mycobacterial model membranes and their interaction with rifabutin: Towards lipid-guided drug screening in tuberculosis. Biochim. Biophys. Acta, Biomembr. 2019, 1861, 1213–1227. 10.1016/j.bbamem.2019.04.004. 31002767

[ref8] Haralampiev I.; et al. The interaction of sorafenib and regorafenib with membranes is modulated by their lipid composition. Biochim. Biophys. Acta 2016, 1858, 2871–2881. 10.1016/j.bbamem.2016.08.014. 27581086

[ref9] Goldman R. C. Why are membrane targets discovered by phenotypic screens and genome sequencing in *Mycobacterium tuberculosis*?. Tuberculosis 2013, 93, 569–588. 10.1016/j.tube.2013.09.003. 24119636

[ref10] Layre E.; et al. A comparative lipidomics platform for chemotaxonomic analysis of *Mycobacterium tuberculosis*. Chem. Biol. 2011, 18, 1537–1549. 10.1016/j.chembiol.2011.10.013. 22195556 PMC3407843

[ref11] Seddon A. M.; et al. Drug interactions with lipid membranes. Chem. Soc. Rev. 2009, 38, 2509–2519. 10.1039/b813853m. 19690732

[ref12] Lopes D.; et al. Shedding light on the puzzle of drug-membrane interactions: Experimental techniques and molecular dynamics simulations. Prog. Lipid Res. 2017, 65, 24–44. 10.1016/j.plipres.2016.12.001. 27939295

[ref13] Alves A. C.; et al. The daunorubicin interplay with mimetic model membranes of cancer cells: A biophysical interpretation. Biochim. Biophys. Acta, Biomembr. 2017, 1859, 941–948. 10.1016/j.bbamem.2017.01.034. 28153496

[ref14] Alves A. C.; et al. Influence of doxorubicin on model cell membrane properties: insights from in vitro and in silico studies. Sci. Rep. 2017, 7, 6343 10.1038/s41598-017-06445-z. 28740256 PMC5524714

[ref15] Peetla C.; Stine A.; Labhasetwar V. Biophysical interactions with model lipid membranes: applications in drug discovery and drug delivery. Mol. Pharmaceutics 2009, 6, 1264–1276. 10.1021/mp9000662. PMC2757518 19432455

[ref16] Beatty W. L.; et al. Trafficking and release of mycobacterial lipids from infected macrophages. Traffic 2000, 1, 235–247. 10.1034/j.1600-0854.2000.010306.x. 11208107

[ref17] Chiaradia L.; et al. Dissecting the mycobacterial cell envelope and defining the composition of the native mycomembrane. Sci. Rep. 2017, 7, 12807 10.1038/s41598-017-12718-4. 28993692 PMC5634507

[ref18] Sani M.; et al. Direct visualization by cryo-EM of the mycobacterial capsular layer: a labile structure containing ESX-1-secreted proteins. PLoS Pathog. 2010, 6, e1000794 10.1371/journal.ppat.1000794. 20221442 PMC2832766

[ref19] Hoffmann C.; et al. Disclosure of the mycobacterial outer membrane: cryo-electron tomography and vitreous sections reveal the lipid bilayer structure. Proc. Natl. Acad. Sci. U.S.A. 2008, 105, 3963–3967. 10.1073/pnas.0709530105. 18316738 PMC2268800

[ref20] Sartain M. J.; et al. Lipidomic analyses of *Mycobacterium tuberculosis* based on accurate mass measurements and the novel ″Mtb LipidDB″. J. Lipid Res. 2011, 52, 861–872. 10.1194/jlr.M010363. 21285232 PMC3073466

[ref21] Ortalo-Magné A.; et al. Identification of the surface-exposed lipids on the cell envelopes of *Mycobacterium tuberculosis* and other mycobacterial species. J. Bacteriol. 1996, 178, 456–461. 10.1128/jb.178.2.456-461.1996. 8550466 PMC177678

[ref22] Pinheiro M.; Pereira-Leite C.; Arêde M.; Nunes C.; Caio J. M.; Moiteiro C.; Giner-Casares J. J.; Lúcio M.; Brezesinski G.; Camacho L.; Reis S. Evaluation of the Structure–Activity Relationship of Rifabutin and Analogs: A Drug–Membrane Study. ChemPhysChem 2013, 14, 2808–2816. 10.1002/cphc.201300262. 23821530

[ref23] Kaiser R. D.; London E. Location of diphenylhexatriene (DPH) and its derivatives within membranes: comparison of different fluorescence quenching analyses of membrane depth. Biochemistry 1998, 37, 8180–8190. 10.1021/bi980064a. 9609714

[ref24] Lakowicz J. R. Principles of Fluorescence Spectroscopy; Springer Science & Business Media, 2013.

[ref25] Howard N. C.; et al. *Mycobacterium tuberculosis* carrying a rifampicin drug resistance mutation reprograms macrophage metabolism through cell wall lipid changes. Nat. Microbiol. 2018, 3, 1099–1108. 10.1038/s41564-018-0245-0. 30224802 PMC6158078

[ref26] Lahiri N.; et al. Rifampin Resistance Mutations Are Associated with Broad Chemical Remodeling of *Mycobacterium tuberculosis*. J. Biol. Chem. 2016, 291, 14248–14256. 10.1074/jbc.M116.716704. 27226566 PMC4933180

[ref27] Villeneuve M.; Kawai M.; Kanashima H.; Watanabe M.; Minnikin D. E.; Nakahara H. Temperature dependence of the Langmuir monolayer packing of mycolic acids from *Mycobacterium tuberculosis*. Biochim. Biophys. Acta, Biomembr. 2005, 1715, 71–80. 10.1016/j.bbamem.2005.07.005. 16125133

[ref28] Vostrikov V. V.; Selishcheva A. A.; Sorokoumova G. M.; Shakina Y. N.; Shvets V. I.; Savel’ev O. Y.; Polshakov V. I. Distribution coefficient of rifabutin in liposome/water system as measured by different methods. Eur. J. Pharm. Biopharm. 2008, 68, 400–405. 10.1016/j.ejpb.2007.05.014. 17614265

[ref29] Zorila B.; Bacalum M.; Popescu A. I.; Radu M. Log-normal deconvolution of laurdan fluorescence spectra—A tool to assess lipid membrane fluidity. Rom. Rep. Phys. 2016, 68, 702–712.

[ref30] Adhyapak P.; Dong W.; Dutta A.; Duan M.; Kapoor S. Lipid Clustering within Mycobacterial Cell Envelope Layers Governs Spatially Resolved Solvation Dynamics Chem. - Asian J., 2022,provisionally accepted. 10.1002/asia.202200146 35419975

[ref31] Tsuchiya H.; Mizogami M. Interaction of drugs with lipid raft domains asa possible target. Drug Target Insights 2020, 14, 34–47. 10.33393/dti.2020.2185. 33510571 PMC7832984

[ref32] Ladavière C.; Gref R. Toward an optimized treatment of intracellular bacterial infections: input of nanoparticulate drug delivery systems. Nanomedicine 2015, 10, 3033–3055. 10.2217/nnm.15.128. 26420270

[ref33] Dulberger C. L.; Rubin E. J.; Boutte C. C. The mycobacterial cell envelope - a moving target. Nat. Rev. Microbiol. 2020, 18, 47–59. 10.1038/s41579-019-0273-7. 31728063

[ref34] Smeulders M. J.; Keer J.; Speight R. A.; Williams H. D. Adaptation of *Mycobacterium smegmatis* to stationary phase. J. Bacteriol. 1999, 181, 270–283. 10.1128/JB.181.1.270-283.1999. 9864340 PMC103559

[ref35] Blokpoel M. C.; Smeulders M. J.; Hubbard J. A.; Keer J.; Williams H. D. Global analysis of proteins synthesized by *Mycobacterium smegmatis* provides direct evidence for physiological heterogeneity in stationary-phase cultures. J. Bacteriol. 2005, 187, 6691–6700. 10.1128/JB.187.19.6691-6700.2005. 16166531 PMC1251579

[ref36] Mathew R.; Ojha A.; Karande A. A.; Chatterji D. Deletion of the rel gene in *Mycobacterium smegmatis* reduces its stationary phase survival without altering the cell-surface associated properties. Curr. Sci. 2004, 149–153.

[ref37] Bansal-Mutalik R.; Nikaido H. Quantitative lipid composition of cell envelopes of *Corynebacterium glutamicum* elucidated through reverse micelle extraction. Proc. Natl. Acad. Sci. U.S.A. 2011, 108, 15360–15365. 10.1073/pnas.1112572108. 21876124 PMC3174599

[ref38] Kapoor S.; Werkmüller A.; Denter C.; Zhai Y.; Markgraf J.; Weise K.; Opitz N.; Winter R. Temperature–pressure phase diagram of a heterogeneous anionic model biomembrane system: Results from a combined calorimetry, spectroscopy and microscopy study. Biochim. Biophys. Acta, Biomembr. 2011, 1808, 1187–1195. 10.1016/j.bbamem.2011.01.011. 21262194

[ref39] Lee T. H.; Hofferek V.; Sani M. A.; Separovic F.; Reid G.; Aguilar M. I. The Impact of Antibacterial Peptides on Bacterial Lipid Membranes Depends on Stage of Growth. Faraday Discuss. 2021, 232, 399–418. 10.1039/D0FD00052C. 34558564

[ref40] Greenwood A. I.; Tristram-Nagle S.; Nagle J. F. Partial molecular volumes of lipids and cholesterol. Chem. Phys. Lipids 2006, 143, 1–10. 10.1016/j.chemphyslip.2006.04.002. 16737691 PMC2695672

[ref41] Wiener M. C.; Tristram-Nagle S.; Wilkinson D. A.; Campbell L. E.; Nagle J. F. Specific volumes of lipids in fully hydrated bilayer dispersions. Biochim. Biophys. Acta, Biomembr. 1988, 938, 135–142. 10.1016/0005-2736(88)90153-8. 2829963

[ref42] Alves A. C.; Ribeiro D.; Horta M.; Lima J. L.; Nunes C.; Reis S. A biophysical approach to daunorubicin interaction with model membranes: relevance for the drug’s biological activity. J. R. Soc., Interface 2017, 14, 20170408 10.1098/rsif.2017.0408. 28855387 PMC5582131

[ref43] Magalhães L. M.; Nunes C.; Lúcio M.; Segundo M. A.; Reis S.; Lima J. L. High-throughput microplate assay for the determination of drug partition coefficients. Nat. Protoc. 2010, 5, 1823–1830. 10.1038/nprot.2010.137. 21030957

[ref44] Pinheiro M.; Pisco S.; Silva A. S.; Nunes C.; Reis S. Evaluation of the effect of rifampicin on the biophysical properties of the membranes: significance for therapeutic and side effects. Int. J. Pharm. 2014, 466, 190–197. 10.1016/j.ijpharm.2014.03.005. 24607210

[ref45] Fernandes M. X.; de la Torre J. G.; Castanho M. A. Joint determination by Brownian dynamics and fluorescence quenching of the in-depth location profile of biomolecules in membranes. Anal. Biochem. 2002, 307, 1–12. 10.1016/S0003-2697(02)00024-6. 12137772

[ref46] Denicola A.; Souza J. M.; Radi R.; Lissi E. Nitric oxide diffusion in membranes determined by fluorescence quenching. Arch. Biochem. Biophys. 1996, 328, 208–212. 10.1006/abbi.1996.0162. 8638932

[ref47] Moench S. J.; Moreland J.; Stewart D. H.; Dewey T. G. Fluorescence studies of the location and membrane accessibility of the palmitoylation sites of rhodopsin. Biochemistry 1994, 33, 5791–5796. 10.1021/bi00185a017. 8180207

[ref48] Ferreira H.; Lúcio M.; de Castro B.; Gameiro P.; Lima J. L. F. C.; Reis S. Partition and location of nimesulide in EPC liposomes: a spectrophotometric and fluorescence study. Anal. Bioanal. Chem. 2003, 377, 293–298. 10.1007/s00216-003-2089-5. 12898116

[ref49] Neves A. R.; Nunes C.; Amenitsch H.; Reis S. Effects of resveratrol on the structure and fluidity of lipid bilayers: a membrane biophysical study. Soft Matter 2016, 12, 2118–2126. 10.1039/C5SM02905H. 26745787

[ref50] Lakowicz J. R. Fluorescence Anisotropy. Principles of Fluorescence Spectroscopy; Springer: Boston, MA, 1999; pp 291–319.

[ref51] Steiner R. F. Fluorescence Anisotropy: Theory and Applications. Topics in Fluorescence Spectroscopy; Springer: Boston, MA, 2002; pp 1–52.

[ref52] Marczak A. Fluorescence anisotropy of membrane fluidity probes in human erythrocytes incubated with anthracyclines and glutaraldehyde. Bioelectrochemistry 2009, 74, 236–239. 10.1016/j.bioelechem.2008.11.004. 19064336

[ref53] Pinheiro M.; Arêde M.; Caio J. M.; Moiteiro C.; Lúcio M.; Reis S. Drug–membrane interaction studies applied to N′-acetyl-rifabutin. Eur. J. Pharm. Biopharm. 2013, 85, 597–603. 10.1016/j.ejpb.2013.02.015. 23523541

[ref54] Pinheiro M.; Silva A. S.; Reis S. Molecular interactions of rifabutin with membrane under acidic conditions. Int. J. Pharm. 2015, 479, 63 10.1016/j.ijpharm.2014.12.042. 25542991

[ref55] Jay A. G.; Hamilton J. A. Disorder amidst membrane order: standardizing laurdan generalized polarization and membrane fluidity terms. J. Fluoresc. 2017, 27, 243–249. 10.1007/s10895-016-1951-8. 27738919

[ref56] Bacalum M.; Zorilă B.; Radu M. Fluorescence spectra decomposition by asymmetric functions: Laurdan spectrum revisited. Anal. Biochem. 2013, 440, 123–129. 10.1016/j.ab.2013.05.031. 23747535

[ref57] Markey F. Principles of Surface Plasmon Resonance. Real-Time Analysis of Biomolecular Interactions; Springer: Tokyo, 2000; pp 13–22.

[ref58] Nunes C.; Brezesinski G.; Lopes D.; Lima J. L.; Reis S.; Lúcio M. Lipid–drug interaction: biophysical effects of tolmetin on membrane mimetic systems of different dimensionality. J. Phys. Chem. B 2011, 115, 12615–12623. 10.1021/jp206013z. 21936545

[ref59] Träger J.; Widder K.; Kerth A.; Harauz G.; Hinderberger D. Effect of cholesterol and myelin basic protein (MBP) content on lipid monolayers mimicking the cytoplasmic membrane of myelin. Cells 2020, 9, 529 10.3390/cells9030529. PMC7140459 32106542

[ref60] Dennington R.; Keith T. A.; Millam J. M. GaussView, version 6.1; Semichem Inc.: Shawnee Mission, 2016.

[ref61] Dickson C. J.; et al. Lipid14: The Amber Lipid Force Field. J. Chem. Theory Comput. 2014, 10, 865–879. 10.1021/ct4010307. 24803855 PMC3985482

[ref62] Bayly C. I.; et al. A well-behaved electrostatic potential based method using charge restraints for deriving atomic charges: the RESP model. J. Phys. Chem. A 1993, 97, 10269 10.1021/j100142a004.

[ref63] Wang J.; et al. Automatic atom type and bond type perception in molecular mechanical calculations. J. Mol. Graphics Modell. 2006, 25, 247–260. 10.1016/j.jmgm.2005.12.005. 16458552

[ref64] Wang J.; et al. Development and testing of a general amber force field. J. Comput. Chem. 2004, 25, 1157–1174. 10.1002/jcc.20035. 15116359

[ref65] Martínez L.; et al. PACKMOL: a package for building initial configurations for molecular dynamics simulations. J. Comput. Chem. 2009, 30, 2157–2164. 10.1002/jcc.21224. 19229944

[ref66] Pal S.; Balasubramanian S.; Bagchi B. Dynamics of bound and free water in an aqueous micellar solution: analysis of the lifetime and vibrational frequencies of hydrogen bonds at a complex interface. Phys. Rev. E: Stat., Nonlinear, Soft Matter Phys. 2003, 67, 061502 10.1103/PhysRevE.67.061502. 16241228

[ref67] Case D. A.; et al. The Amber biomolecular simulation programs. J. Comput. Chem. 2005, 26, 1668–1688. 10.1002/jcc.20290. 16200636 PMC1989667

[ref68] Ester M. In A Density-Based Algorithm for Discovering Clusters in Large Spatial Databases with Noise, KDD-96, 1996; pp 226–231.

