# Supplementary material for: Mutually Exclusive Interactions of Rifabutin with Spatially Distinct Mycobacterial Cell Envelope Membrane Layers Offer Insights into Membrane-Centric Therapy of Infectious Diseases
Source: ACS Bio Med Chem Au. 2022 Mar 24;2(4):395–408. doi: 10.1021/acsbiomedchemau.2c00010 (PMC9389580; doi:10.1021/acsbiomedchemau.2c00010)
Supplement: Supplementary file 6 — bg2c00010_si_006.pdf [file bg2c00010_si_006.pdf]

Supplementary Information

Mutually Exclusive Interactions of Rifabutin with Spatially Distinct Mycobacterial Cell Envelope Membrane Layers offer Insights for Membrane-Centric Therapy in Infectious Diseases

Anjana P. Menon[1,2,4], Wanqian Dong[3], Tzong-Hsien Lee[2,4], Marie-Isabel Aguilar[2,4,*], Mojie Duan[3*], Shobhna Kapoor[1,2,5*]

[1]*Department of Chemistry, Indian Institute of Technology Bombay, Mumbai 400076, India*

[2]*IITB-Monash Academy, Indian Institute of Technology Bombay, Mumbai 400076, India*

[3] *Innovation Academy for Precision Measurement Science and Technology, Chinese Academy of Sciences, Wuhan 430071, China*

[4]*Department of Biochemistry & Molecular Biology, Monash University, Clayton, Vic 3800, Australia*

[5]*Graduate School of Integrated Sciences for Life, Hiroshima University, Hiroshima, 739-8528, Japan*

This supplementary file contains SI text, and 7 SI figures, and 11 SI Tables

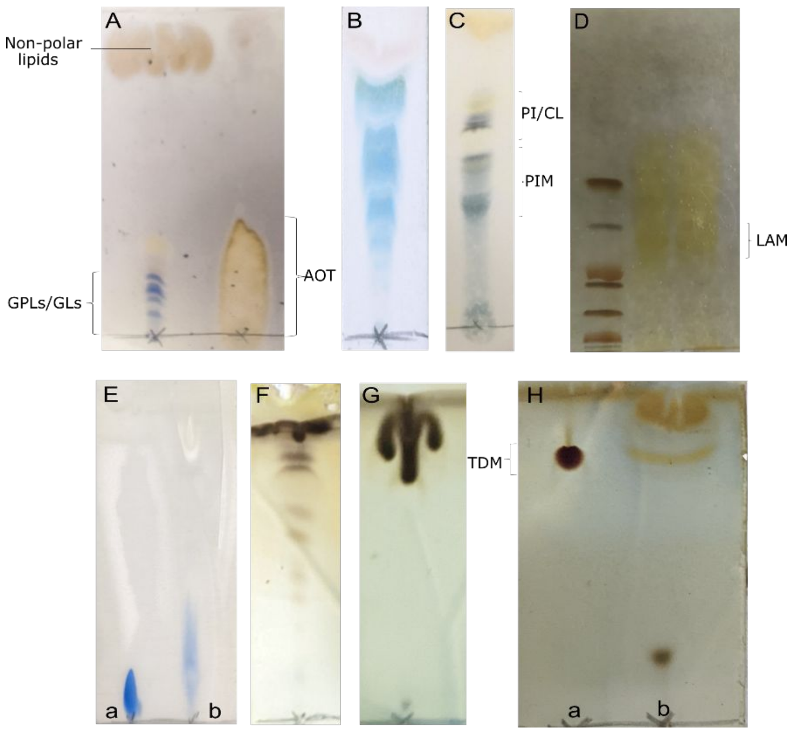

Fig. S1: (A-C) TLC profiles of extracted lipids developed using 1% anthrone spray. (A) TLC profile of outer membrane lipids after (purified) and before AOT separation developed in chloroform-methanol mixture (C:M) (9:1). (B) Purified outer membrane lipids TLC developed in 3x C:M (9.5:0.5) indicates the density of GPLs and glycolipids within the mixture. (C) Glycolipids in the inner membrane lipids were visualized using anthrone spray and developed in a mixture of CMW (65:25:1). (D) SDS PAGE profile of LAM indicates that its size is ~40kD.(E) TLC profiles of (b) IML developed using phosphospray. (a) DPPC is used to compare the phospholipids detected in the IML. (F-H) TLC profiles of extracted lipids developed using phosphospray followed by charring to detected mycolic acids. (F) Distribution of mycolic acids in OML. (Hb) Distribution of mycolic acids in PAL. (G) Standard mycolic acid mixture and (Ha) TDM is used as a reference to mycolic acid detection.

Table S1:List of lipids identified through LCMS in the outer, inner and peptidoglycan-associated fractions, using the online mycobacterial databases, LipidDB and MycoMass [1].

Outer Membrane Lipids	Inner Membrane Lipids	Peptidoglycan-Associated Lipids
Ac2SGL (C56)	Ac1PIM1 (R1CO2H+R2CO2H+R3CO2H =55:3)	CL (2R1CO2H+2R2CO2H= 62:0)
Alpha-MA (C89)	Ac1PIM1 (R1CO2H+R2CO2H+R3CO2H =55:5)	DAT (C27:0)
Carboxylic exochelins threonine	Ac1PIM2 (R1CO2H+R2CO2H+R3CO2H =51:3,R4=H)	Lyso-PE (R1CO2H=16:0)
DAT (C25:2)	Ac1PIM2 (R1CO2H+R2CO2H+R3CO2H =54:2,R4=H)	Lyso-PE (R1CO2H=17:0)
DAT (C26:0)	Ac1PIM2 (R1CO2H+R2CO2H+R3CO2H=49:0,R4=H)	Lyso-PE (R1CO2H=18:0)

DAT (C27:0)	Ac1PIM2 (R1CO2H+R2CO2H+R3CO2H=50:2,R4=H)	Lyso-PE (R1CO2H=19:1)
DAT (C32:0)	Ac1PIM3 (R1CO2H+R2CO2H+R3CO2H =50:0,R4=H)	Lyso-PE (R1CO2H=20:0)
DAT (C37:1)	Ac1PIM3 (R1CO2H+R2CO2H+R3CO2H=47:1,R4=H)	Lyso-PG (RCO2H=16:0)
DAG (R1CO2H+R2CO2H=30:1)	Ac1PIM3 (R1CO2H+R2CO2H+R3CO2H=47:1,R4=H)	Lyso-PG (RCO2H=18:0)
DAG (R1CO2H+R2CO2H=32:2)	Ac1PIM3 (R1CO2H+R2CO2H+R3CO2H=50:0,R4=H)	Lyso-PG (RCO2H=19:1)
Dideoxy Mycobactins (C18:1)	CM (Carboxymycobactins)	Lyso-PG (RCO2H=20:0)
Glycosylated phthiodiolone dimycocerosate (C106)	DAG (C24:2)	Lyso-PI (RCO2H=18:0)
GPL-IIa/IV	DAG (C25:0)	Lyso-PI (RCO2H=18:1)
GPL-IIb	DDCM (dideoxycarboxymycobactin methylesters) (C8:1)	Lyso-PI (RCO2H=18:1)
GPL-serovar 1	DDCM (dideoxycarboxymycobactin methylesters) (C12:1)	Lyso-PI (RCO2H=19:0)
Hydroxyphthioceranic acid (C34)	DDCM (serine butyric acid dideoxycarboxymycobactins) (C9:1)	MG (RCO2H=14:0)
Keto-MA (C83)	DDCM (threonine/a-methyl serine ethylbutyric acid dideoxycarboxymycobactins) (C6:1)	MG (RCO2H=16:0)
Lysinated PG (C20:0)	L5P (lipopentapeptide)	MG (RCO2H=16:1)
Lyso PA (C20:4)	L5P (Lipopentides)	MPM (C32)
Lyso PE (C30:1)	LPA (C16:0)	Mycolipanolic acid (C27)
Lyso PG (C14:0)	LPA (Lysophosphatidic Acid) (C20:4)	PE(R1CO2H+R2CO2H=31:0)
Lyso PG (C16:0)	LPE (C22:0)	PE(R1CO2H+R2CO2H=33:1)
Lyso phosphatidic acid (C29:1)	LPE (C24:1)	PE (R1CO2H+R2CO2H=36:0)
Lyso PI (C24:0)	LPE (C29:1)	PE (R1CO2H+R2CO2H=36:2)
Lyso-PA C18:2)	LPG (C16:0)	PG (R1CO2H+R2CO2H=30:2)
Lyso-PE (R1CO2H=16:1)	LPG (C23:1)	PG (R1CO2H+R2CO2H=31:0)
Lyso-PE (R1CO2H=18:0)	LPI (C24:0)	PG (R1CO2H+R2CO2H=31:2)
Lyso-PE (R1CO2H=19:1)	LPI (C24:1)	PG (R1CO2H+R2CO2H=37:0)
Lyso-PG (C17:1)	LPI (Lyso phosphatidylinositols) (C13:1)	PG(R1CO2H+R2CO2H =30:2)

Lyso-PG (C19:0)	LPI (Lyso phosphatidylinositols) (C27:1)	PG(R1CO2H+R2CO2H =36:2)
Lyso-PG (C21:0)	LPI (Lysophospholipids) (C21:1)	PG(R1CO2H+R2CO2H =37:0)
Lyso-PG (C23:1)	Lyso PA (20:4)	PI (R1CO2H+R2CO2H=33: 2)
Lyso-PG (C24:1)	Lyso-PE (R1CO2H=19:0)	PI (R1CO2H+R2CO2H=34: 1)
Lyso-PG (RCO2H=15:1)	Lyso-PG (RCO2H=17:0)	PI (R1CO2H+R2CO2H=37: 1)
Lyso-PG (RCO2H=18:1)	Lyso-PG (RCO2H=19:1)	PI (R1CO2H+R2CO2H=42: 1)
Lyso-PG (RCO2H=19:0)	Lyso-PI (RCO2H=17:0)	PIM1(R1CO2H+R2CO2H =32:1)
Menaquinone MK-5 (II-H2) (C25:4)	Lyso-PI (RCO2H=18:1)	PIM1(R1CO2H+R2CO2 H=37:0)
MG (RCO2H=14:0)	Mbt +Fe (R=17:0)	Hydroxyphthioceranic acid (C39)
MG (RCO2H=15:0)	Mbt +Fe (R=17:1)	Hydroxyphthioceranic acid (C40)
MG (RCO2H=16:1)	MDMB (Monodeoxymycobactins (C22:0)	Mycocerosic acid (C30)
MG (RCO2H=18:0)	MDMB (serine butyric acid monodeoxymycobactins)	Mycocerosic acid (C32)
MG (RCO2H=21:0)	MDMB(Non-ribosomal peptide/polyketide hybrids) (C19:1)	Mycocerosic acid (C35)
MG (RCO2H=22:0)	MK-6 (Menaquinones) (C30:6)	DG (R1CO2H+R2CO2H=33: 0)
MPM (C30)	MMDAG (Meromycolyl diacylglycerols) (C76:1)	DAG (R1CO2H+R2CO2H=34: 2)
MPM (C32)	MPM (C32)	Mycolipanolic acid (C26)
MPM (C33)	PA (C29:2)	Mycolipenic acid (C27)
MPM (C34)	PA (C30:2)	Mycolipenic acid (C28)
Mycobactins (C22:1)	PA (C33:0)	Mycolipenic acid (C29)
Mycocerosic acid (C27)	PA (C49:2)	Mycosanoic or Mycocerosic acid (C24)
Mycocerosic acid (C32)	PA (Phosphatdic Acid) (C29:1)	Phthioceranic acid (C36)
Mycolipanolic acid (C28)	PA (Phosphatdic Acid) (C30:2)	Phthioceranic acid (C45)
Mycosanoic or Mycocersoci acid(C26)	PA (Phosphatdic Acid) (C32:1)	
PA (C25)	PA (Phosphatdic Acid) (C33:1)	
PA (C25:0)	PA (Phosphatdic Acid) (C49:2)	
PA (C25:1)	PA (Phosphatidic Acid) (C29:1)	
PA (C25:2)	PA (Phosphatidic Acid) (C32:1)	
PA (C28:1)	PDIM A (C56:0)	
PA (C29:1)	PE (25:1)	
PA (C29:2)	PE (30:2)	
PA (C30:2)	PE (C 34:0)	
PA (C31)	PE (C 48:0)	
PA (C31:2)	PE (C25:1)	
PA (C32:1)	PE (C25:1)	
PA (C33:1)	PE (C25:2)	

PA (C33:2)	PE (C25:2)	
PA (C34:1)	PE (C26:0)	
PA (C46:1)	PE (C28:0)	
PE (C28:1)	PE (C28:1)	
PE (C31:0)	PE (C28:1)	
PE (C31:1)	PE (C29:1)	
PE (R1CO2H+R2CO2H=27:1)	PE (C30:2)	
PE (R1CO2H+R2CO2H=28:1)	PE (C31:0)	
PE (R1CO2H+R2CO2H=29:1)	PE (C31:1)	
PE (R1CO2H+R2CO2H=30:0)	PE (C31:2)	
PE (R1CO2H+R2CO2H=30:1)	PE (C32:0)	
PE (R1CO2H+R2CO2H=31:0)	PE (C34:0)	
PE (R1CO2H+R2CO2H=31:1)	PE (R1CO2H+R2CO2H=31:0)	
PE (R1CO2H+R2CO2H=32:2)	PE (R1CO2H+R2CO2H=33:1)	
PE (R1CO2H+R2CO2H=33:0)	PE (R1CO2H+R2CO2H=36:0)	
PE (R1CO2H+R2CO2H=33:1)	PE (R1CO2H+R2CO2H=36:2)	
PE (R1CO2H+R2CO2H=34:1)	PG (26:0)	
PE (R1CO2H+R2CO2H=35:1)	PG (C24:2)	
PE (R1CO2H+R2CO2H=35:2)	PG (C26:0)	
PE (R1CO2H+R2CO2H=36:2)	PG (C26:1)	
PE (R1CO2H+R2CO2H=39:1)	PG (C28:2)	
PG (C24:0)	PG (C31:2)	
PG (C24:1)	PG (R1CO2H+R2CO2H=31:2)	
PG (C27:0)	PI (C 47:0)	
PG (C29:1)	PI (C24:2)	
PG (C30:2)	PI (C25:0)	
PG (C31:2)	PI (C25:2)	
PG (C32:2)	PI (C25:2)	
PG (R1CO2H+R2CO2H=24:0)	PI (C33:1)	
PG (R1CO2H+R2CO2H=24:1)	PI (R1CO2H+R2CO2H=32:2)	
PG (R1CO2H+R2CO2H=28:0)	PI (R1CO2H+R2CO2H=33:1)	
PG (R1CO2H+R2CO2H=28:2)	PIM3 (R1CO2H+R2CO2H=31:0)	
PG (R1CO2H+R2CO2H=29:1)	PIM4 (R1CO2H+R2CO2H=34:1)	
PG (R1CO2H+R2CO2H=30:2)	TAT (Triacyltrehalose)	
PG (R1CO2H+R2CO2H=31:1)	TAT (Triacyltrehalose) (49:0)	
PG (R1CO2H+R2CO2H=31:2)	CL (2R1CO2H+2R2CO2H=58:2)	
PG (R1CO2H+R2CO2H=33:2)	CL (2R1CO2H+2R2CO2H=59:3)	
PG (R1CO2H+R2CO2H=34:1)	CL (2R1CO2H+2R2CO2H=59:3)	
PG (R1CO2H+R2CO2H=36:2)	PIM1 (R1CO2H+R2CO2H=34:1)	
PG (R1CO2H+R2CO2H=37:0)	PIM2 (R1CO2H+R2CO2H=35:0)	
PG C36:0	PIM3 (R1CO2H+R2CO2H=30:0)	
Phosphatidic acid PA (C25:2)	PIM3 (R1CO2H+R2CO2H=33:1)	
Phosphatidic acids (28:1)	PIM3 (R1CO2H+R2CO2H=36:0)	
Phosphatidic acids (C32:1)	PIM6 (R1CO2H+R2CO2H=30:0)	
Phthioceranic acid (C36)		
PI (C36:2)		
PI (R1CO2H+R2CO2H=32:2)		
PI (R1CO2H+R2CO2H=33:1)		
PI (R1CO2H+R2CO2H=34:1)		
PI (R1CO2H+R2CO2H=34:1)		
PI (R1CO2H+R2CO2H=36:1)		
PIM1 (R1CO2H+R2CO2H=37:2)		
SL-II OR SL-II'(C167)		
SL-III		
TDM (C169)		
TG(R1CO2H+R2CO2H+R3CO		

2H=73:1)		
Mycolipenic acid (C27)		
Mycolipenic acid (C28)		
Mycolipenic acid (C29)		

Table S2: The average order parameters (-Scd) of the carbon atoms of the tails of lipids in mycobacterial membrane.

Outer/ myco membrane	Tail	$-<S_{cd}>$	
		- Rifabutin	+ Rifabutin
LAM	Tail1: C18	0.30	0.29
	Tail2: C18	0.32	0.34
	Tail3: C18	0.32	0.33
MA	Tail1 : 25:0	0.35	0.35
	Tail2 : 37:1	0.32	0.32
SL-1	Tail1 : 16:0	0.36	0.34
	Tail2 : C33	0.34	0.34
	Tail3 : C32	0.34	0.34
	Tail4 : C32	0.33	0.33
TDM	Tail1 : C26	0.33	0.33
	Tail2 : C31	0.28	0.29
	Tail3 : C25	0.30	0.29
	Tail4 : C32	0.29	0.30
PDIM	Tail1 : C22	0.16	0.16
	Tail2 : C27	0.22	0.23
	Tail3 : C27	0.24	0.23
DOPC	Tail1 : 18:1	0.17	0.16
	Tail2 : 18:1	0.20	0.19
DPPC	Tail1 : 16:0	0.35	0.35
	Tail2 : 16:0	0.36	0.37

Inner Membrane	Tail	$-<S_{cd}>$	
		- Rifabutin	+ Rifabutin
Ac2PIM	Tail1 : 16:0	0.26	0.26
	Tail2 : C18	0.23	0.23
CL	Tail1 : 14:0	0.23	0.22
	Tail2 : 16:0	0.25	0.25
	Tail3 : 14:0	0.25	0.24
	Tail4 : 18:1	0.20	0.20
DG	Tail1 : 18:1	0.28	0.28
	Tail2 : 16:0	0.34	0.34
PE	Tail1 : 18:1	0.25	0.27
	Tail2 : 16:0	0.32	0.33
PG	Tail1 : 18:1	0.20	0.21
	Tail2 : 16:0	0.25	0.26
PI	Tail1 : 18:1	0.24	0.23
	Tail2 : 16:0	0.28	0.28

Table S3: Comparison of total lipid fractions from Mycobacterium smegmatis [1] from Mycobacterium tuberculosis [2].

Mycobacterium tuberculosis (Mtb)	***Mycobacterium smegmatis (Msm)***
PDIM A	PDIM A
PDIM B	PDIM B
Phthiotriol DIM	
	Glycosylated Phthiotriol DIM
Alpha-MA	Alpha-MA
Keto-MA	Keto-MA
Methoxy-MA	Methoxy-MA
TAG	TAG
DAG	DAG
Glucuronosyl DAG	
Diglycosylated DAG	
Monomeromycolyl DAG	Monomeromycolyl DAG
MAG	MAG

Menaquinones	Menaquinones
Sulfomenaquinones	
Mycobactins	Mycobactins
Monodeoxymycobactins	
Dideoxymycobactins	dideoxymycobactins
	carboxymycobactins
dideoxycarboxymycobactins	dideoxycarboxymycobactins
	serinebutyric acid dideoxycarboxymycobactins
monodeoxycarboxymycobactins	
Alpha-glycerol monomycolate	Alpha-glycerol monomycolate
Keto-glycerol monomycolate	Keto-glycerol monomycolate
Methoxy-glycerol monolmycolate	Methoxy-glycerol monolmycolate
Alpha-glucose monomycolate	
Methoxy-glucose monomycolate	
Leprosol	Leprosol
Mycosanoic acids	Mycosanoic acids
Alpha-TMM	
Keto-TMM	
Methoxy-TMM	
TDM	TDM
TAT	TAT
DAT	DAT
Lipopentapeptide	Lipopentapeptide
PI	PI
Lyso-PI	Lyso-PI
PE	PE
Lyso-PE	Lyso-PE
PG	PG
	LysoPG
PA	PA
Lyso-PA	Lyso-PA
Decaprenylphosphoribose	Decaprenylphosphoribose
Cardiolipins	Cardiolipins
Tetraacylated sulfoglycolipids	Tetraacylated sulfoglycolipids
Diacylated sulfoglycolipids	Diacylated sulfoglycolipids
SL I	
SL II	SL II
SL III	SL III
	PIM1
	PIM2
	PIM3
	PIM4
	PIM6
Monoacyl PIM	Monoacyl PIM
Diacyl PIM	Diacyl PIM
	Glycosylated PIM
Monoglycosyl parahydroxybenzoic acid derivatives	
Hydroxyphthioceranic acids	Hydroxyphthioceranic acids
Mycoketidesmannosyl phospho	
	Carboxylic exochelins threonine
	GPL
	Mycolipanoic acid
	Mycolipenic acid

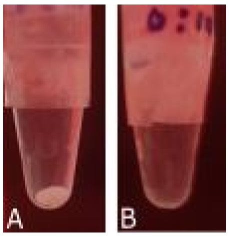

Figure S2: Neutral buoyancy method for the determination of specific volume of the lipids to finally calculate molar volume (V_m). In vial **A**, pure H_2O is less dense than the lipids, hence the lipids sink. In vial **B**, pure D_2O is used, which is denser than the lipids, and hence no lipids sink.

Table S4: Molar volumes (V_m) of different lipid mixtures (L/mol) derived by the neutral buoyancy method.

	Outer Membrane	Inner Membrane	Mycomembrane
V_m	1.13	1.5	1.25

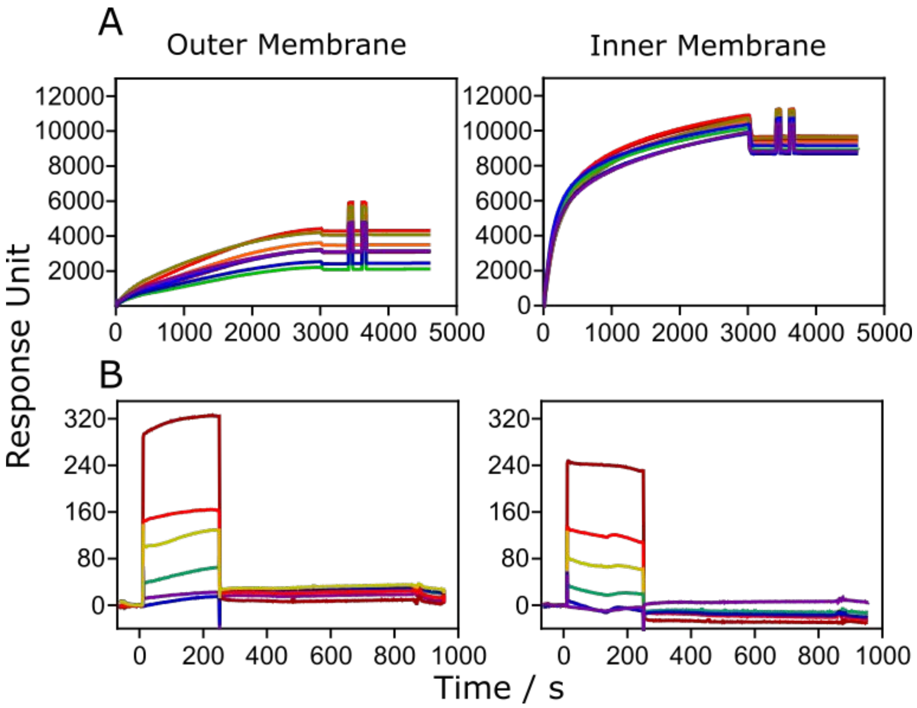

Figure S3: Surface plasmon resonance sensorgrams of Rifabutin-mycobacterial membrane interactions. (A) Sensorgrams of lipid deposition onto the L1 chip. (B) Interaction of different concentrations (0 - 60 μM) of Rifabutin with the outer (left) and inner (right) mycobacterial systems.

Table S5: The average number of Rifabutin molecules (×100) in contact with a single lipid. The highest and lowest interaction values for each mycobacterial membrane is highlighted in blue and red respectively.

Mycomembrane	LAM	MA	PDIM	SL-1	TDM	DOPC	DPPC
	1.26	0.32	0.54	0.63	0.89	0.21	0.16
Inner membrane	Ac2PIM	Cardiolipin	DAG	PE	PG	PI	
	0.16	0.22	0.0	0.04	0.0	0.01	

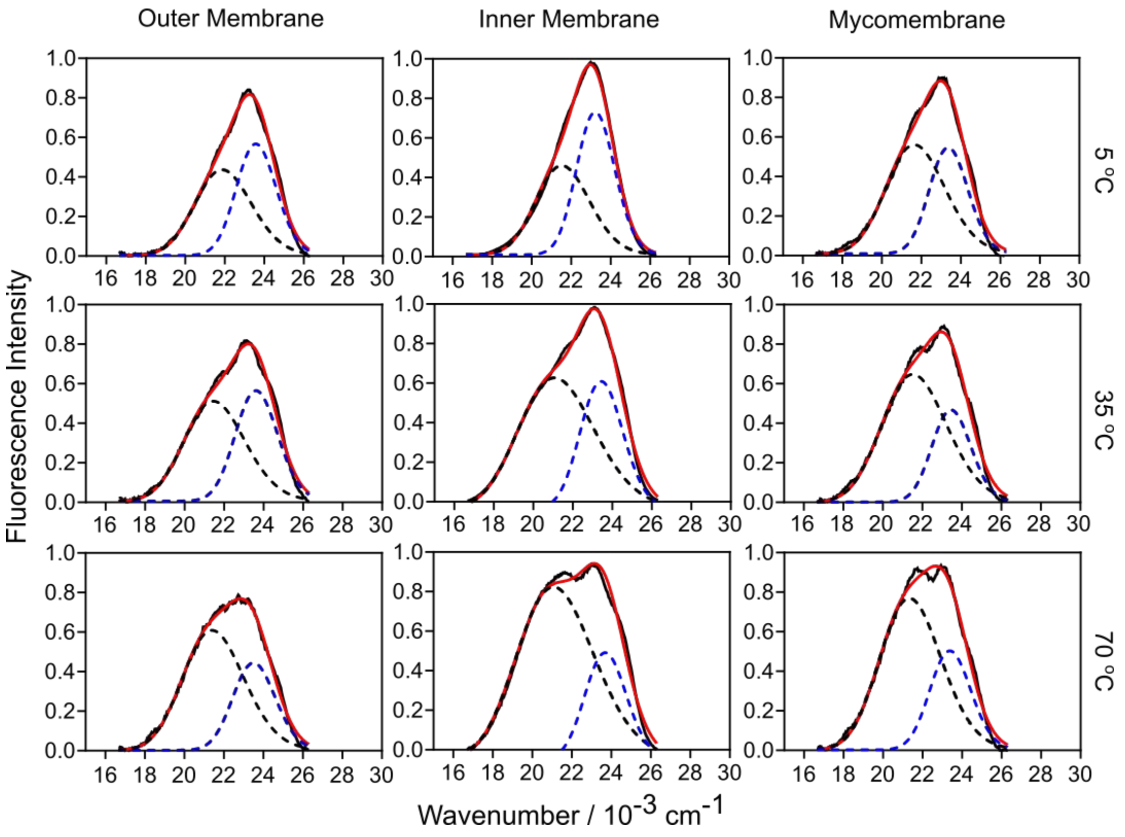

Figure S4: Laurdan deconvolution of the normalized and baseline corrected fluorescence intensity curves (solid black line) into charge-transfer states (black dashes) and solvent-relaxed state (blue dashes) by log-normal deconvolution method (red) at three different temperatures for all the *Msm* membrane systems. The deconvoluted curves were used to calculate Laurdan GP values.

Table S6: The fold change in the rigidity of the mycobacterial membranes observed at 60 °C on addition of Rifabutin to the membrane liposomes.

	Outer Membrane	Inner Membrane	Mycomembrane
TMA-DPH	1.38	1.34	1.72
DPH	2.02	1.63	2.30

Table S7. The area distribution of disordered region in 40 GUV/lipid mixture/replicate in confocal imaging of the Rh-DHPE tagged *Msm* system was measured in ImageJ.

Lipid System		% area of less ordered region
	-Rifabutin	65.1 ± 9.0

Outer Membrane	+ Rifabutin	65.1 ± 13.9
Inner Membrane	- Rifabutin	63.3 ± 14.0
	+ Rifabutin	72.9 ± 11.9

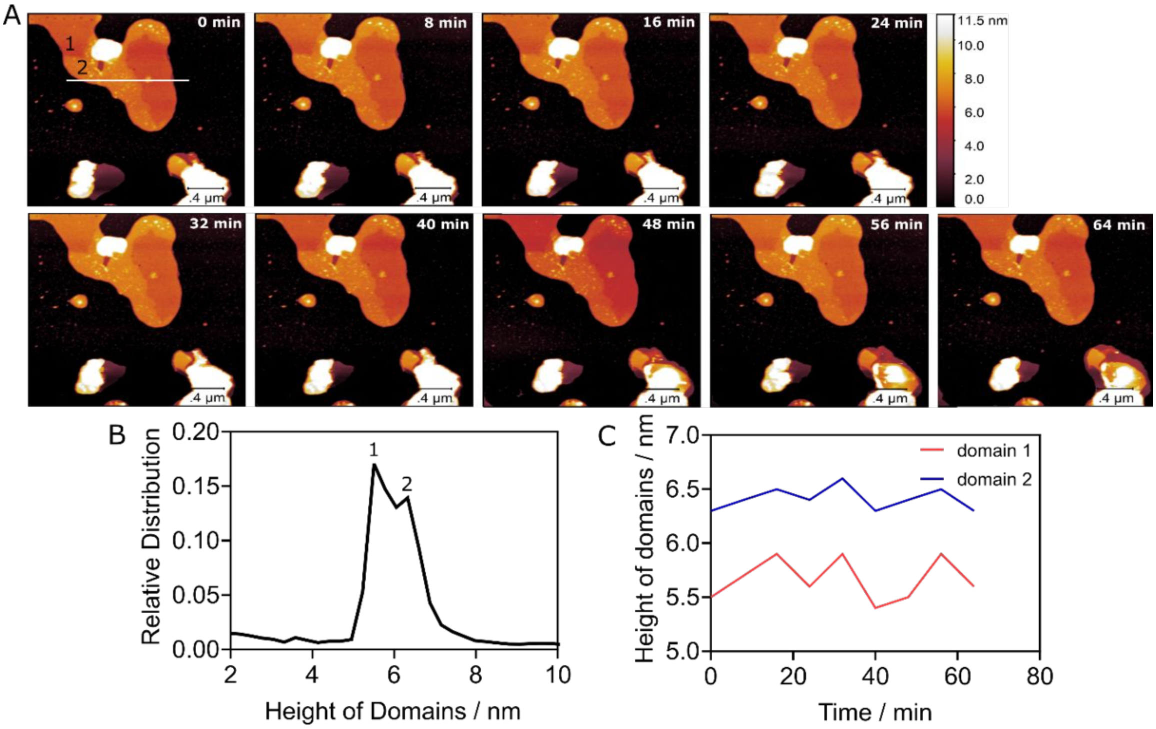

Figure S5: (A) Topography of inner membrane in the absence of Rifabutin observed as solid supported bilayers (SLBs) with atomic force microscopy. (B) The inner membranes displayed at least 2 main domains of ~5.5 nm (domain 1) and ~6.3 nm (domain 2) in height. (C) Temporal changes in the height of the two lipid domains.

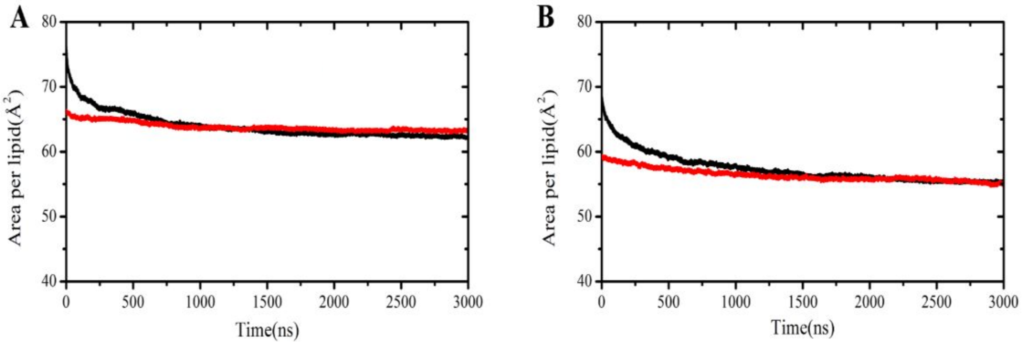

Figure S6: The area per lipid (APL) as a function of simulation time. (A) The area per lipid of myco membrane (black) and in presence of Rifabutin (red) (B) The area per lipid of inner membrane (black) and

in presence of Rifabutin (red). The APL values remain unchanged after 500 ns, indicating the equilibration of the modelled membrane systems.

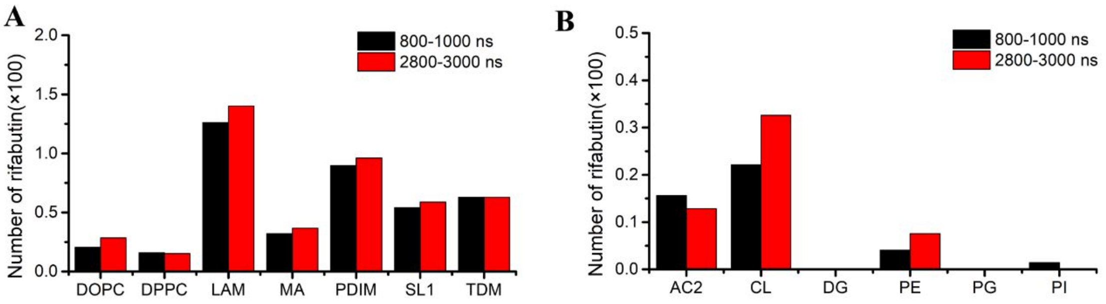

Figure S7: The comparison of rifabutin-lipid contact number in 800-1000 ns and 2800-3000 ns. (A) The contact number of rifabutin with the lipids in the mycomembrane (B) The contact number of rifabutin with the lipids in inner membrane.

Table S8. The average order parameters -<S_{CD}> of the lipids in inner membrane.

Lipid type	Chain	$\langle S_{CD} \rangle$		
		**800-1000 ns**	**2000-2500 ns**	**2500-3000 ns**
Ac2PIM	1	0.26	0.27	0.28
	2	0.23	0.24	0.25
CL	1	0.23	0.24	0.24
	2	0.25	0.27	0.28
	3	0.25	0.26	0.27
	4	0.20	0.23	0.24
DG	1	0.34	0.36	0.37
	2	0.28	0.31	0.32
PE	1	0.32	0.34	0.33
	2	0.25	0.27	0.26
PG	1	0.25	0.26	0.26
	2	0.20	0.21	0.22
PI	1	0.28	0.29	0.29
	2	0.24	0.25	0.25

Table S9. The average order parameters -<S_{CD}> of the lipids in inner membrane in present of rifabutin.

**Lipid type**	**Chain**	$\langle S_{CD} \rangle$		
		**800-1000 ns**	**2000-2500 ns**	**2500-3000 ns**
Ac2PIM	1	0.26	0.27	0.27
	2	0.23	0.24	0.24
CL	1	0.22	0.24	0.23
	2	0.25	0.27	0.28
	3	0.24	0.27	0.27
	4	0.20	0.22	0.22
DG	1	0.34	0.35	0.36
	2	0.28	0.30	0.31
PE	1	0.34	0.35	0.36
	2	0.27	0.28	0.28
PG	1	0.26	0.26	0.27
	2	0.22	0.22	0.22
PI	1	0.28	0.28	0.28
	2	0.23	0.24	0.23

Table S10. The average order parameters -<S_{CD}> of the lipids in mycomembrane.

**Lipid type**	**Chain**	$\langle S_{CD} \rangle$		
		800-1000 ns	2000-2500 ns	2500-3000 ns
DOPC	1	0.17	0.19	0.18
	2	0.21	0.21	0.21
DPPC	1	0.35	0.36	0.36
	2	0.36	0.37	0.38
LAM	1	0.30	0.32	0.32
	2	0.32	0.34	0.35
	3	0.32	0.34	0.35
MA	1	0.35	0.36	0.36
	2	0.32	0.33	0.33
PDIM	1	0.16	0.19	0.19

	2	0.22	0.25	0.25
	3	0.24	0.24	0.25
SL-1	1	0.36	0.37	0.38
	2	0.34	0.36	0.36
	3	0.34	0.34	0.34
	4	0.33	0.34	0.34
TDM	1	0.33	0.34	0.34
	2	0.28	0.29	0.30
	3	0.30	0.30	0.30
	4	0.29	0.30	0.31

Table S11. The average order parameters -<S_{CD}> of the lipids in mycomembrane in presence of Rifabutin.

Lipid type	Chain	$\langle S_{CD} \rangle$		
		**800-1000 ns**	**2000-2500 ns**	**2500-3000 ns**
DOPC	1	0.16	0.16	0.16
	2	0.19	0.20	0.19
DPPC	1	0.35	0.36	0.35
	2	0.37	0.39	0.39
LAM	1	0.29	0.31	0.31
	2	0.34	0.34	0.34
	3	0.33	0.35	0.35
MA	1	0.35	0.36	0.36
	2	0.32	0.33	0.33
PDIM	1	0.16	0.19	0.20
	2	0.23	0.25	0.25
	3	0.23	0.25	0.25
SL1	1	0.34	0.35	0.34
	2	0.34	0.34	0.33
	3	0.34	0.34	0.33
	4	0.33	0.33	0.33
TDM	1	0.33	0.34	0.35

	2	0.29	0.29	0.28
	3	0.29	0.30	0.31
	4	0.30	0.31	0.31

SI References

1. Adhyapak, P., et al., *Dynamical Organization of Compositionally Distinct Inner and Outer Membrane Lipids of Mycobacteria.* Biophys J, 2020. **118**(6): p. 1279-1291.
2. Layre, E., et al., *A comparative lipidomics platform for chemotaxonomic analysis of Mycobacterium tuberculosis.* Chem Biol, 2011. **18**(12): p. 1537-49.
